# From Catering to Clinical Care: An Expert Survey on Designing the Inpatient Psychiatric Meal Concept

**DOI:** 10.3390/nu18152448

**Published:** 2026-07-27

**Authors:** Timur Liwinski, Sandra Nussbaum, Lukas Imfeld, Ulf Wein, Undine Lang

**Affiliations:** 1 Clinic for Adults, University Psychiatric Clinics Basel (UPK Basel), Wilhelm Klein-Strasse 27, 4002 Basel, Switzerland; 2 Faculty of Medicine, University of Basel, Klingelbergstrasse 61, 4056 Basel, Switzerland

**Keywords:** nutritional psychiatry, inpatient psychiatry, hospital catering, meal concept, Mediterranean diet, severe mental illness, dietetics, expert survey

## Abstract

**Background/Objectives**: People with severe mental illness experience high rates of malnutrition and cardiometabolic disease, and hospital food is increasingly regarded as part of clinical care rather than mere catering. Although nutrition is increasingly recognised as a therapeutic component of psychiatric care, little is known about how the professionals closest to nutritional care would design an inpatient meal concept. **Methods**: We conducted a cross-sectional, anonymous online survey of 26 nutrition professionals working in psychiatric settings in the German-speaking D-A-CH region (Germany, Austria, and Switzerland), who rated the goals, patient needs, dietary forms, meal characteristics, organisational aspects, and barriers relevant to an inpatient meal concept on five-point Likert scales. Free-text responses were analysed thematically. **Results**: Respondents prioritised eating enjoyment (mean [*M*] = 4.65) and psychological stability over economic goals, and rated both appetite loss and hyperphagia, together with medication-related metabolic change, as key patient needs. A Mediterranean dietary pattern was clearly the preferred model (*M* = 4.62), whereas low-carbohydrate options were the least endorsed form (*M* = 2.65). Interprofessional communication between nursing, kitchen, and dietetics was the highest-rated item overall (*M* = 4.75), while budget restrictions were seen as the principal barrier. **Conclusions**: In this small, self-selected expert sample, experts largely converged on a patient-centred, Mediterranean, flexible and choice-based meal concept delivered through strong interprofessional processes, supporting a shift from catering toward nutrition as a therapeutic component of inpatient psychiatry.

## 1. Introduction

Over the past decade, the relationship between diet and mental health has moved from the periphery of psychiatric thinking toward a recognised, evidence-informed domain of clinical care. In a consensus position statement, the International Society for Nutritional Psychiatry Research argued that diet is as fundamental to psychiatry as it is to cardiology, endocrinology, and gastroenterology, and called for nutritional medicine to be regarded as mainstream rather than complementary [1]. This reframing is supported by a growing body of epidemiological and interventional evidence linking dietary quality to the risk, course, and treatment of common mental disorders [2,3]. Although the strength of evidence varies by exposure and outcome, meta-analytic syntheses of randomised trials indicate that selected nutrient-based interventions can confer measurable mental-health benefits [4]. In that meta-review of 33 meta-analyses of randomised trials, the strongest evidence was for omega-3 fatty acids—particularly eicosapentaenoic acid—as an adjunctive treatment for depression, with more limited support for most other supplements. Diet, in short, is increasingly understood not as an adjunct to psychiatric treatment but as a modifiable determinant of it.

This conceptual shift has particular implications for the hospital, where food is too often treated as hotel service or catering rather than as clinical care. Disease-related malnutrition is common among hospitalised adults—affecting a substantial proportion of patients already at admission to acute internal-medicine settings—and is consistently associated with prolonged length of stay, greater morbidity, and increased in-hospital mortality [5]. Reported prevalence estimates vary with the population and screening tool, commonly ranging from roughly a fifth to a third of hospitalised patients, and malnutrition independently predicts longer length of stay, more complications, and higher costs [6,7]. Far from being an incidental accompaniment of illness, malnutrition in hospital constitutes a distinct and treatable syndrome linked to impaired recovery, disability, and considerable healthcare costs [7].

Crucially, hospital nutrition is not merely a prognostic marker but a therapeutic target. In the Effect of Early Nutritional Support on Frailty, Functional Outcomes, and Recovery of Malnourished Medical Inpatients (EFFORT) trial—a large, multicentre, randomised study conducted in Switzerland—protocol-guided individualised nutritional support, as opposed to standard hospital food, reduced the risk of adverse clinical outcomes, including mortality, in medical inpatients at nutritional risk [8]. The implication is consequential: delivering the right food to the right patient is itself an intervention, and the meal becomes an instrument of treatment rather than a backdrop.

Among hospitalised populations, psychiatric inpatients are among the most nutritionally vulnerable. People with severe mental illness experience markedly reduced life expectancy, driven in large part by cardiometabolic disease [9], and they exhibit poorer diet quality and more adverse eating behaviours than the general population [10,11]. These disparities are compounded by the disorders themselves and their treatment: psychotropic medication can alter appetite and metabolism [12,13], while symptoms such as appetite loss, hyperphagia, cognitive impairment, and heightened sensory sensitivity may each undermine adequate and balanced intake. Importantly, this vulnerability is modifiable—nutrition and dietary interventions have been shown to improve metabolic risk factors in this population [14]. Evidence from Swiss psychiatric services reinforces both the problem and its tractability: patients requiring intensive psychiatric treatment carry a substantial risk of malnutrition [15], display poorer nutritional status and more disordered and unhealthy eating patterns than healthy adults [16], and face specific, hitherto under-examined barriers to healthy eating within mental-health care [17].

To date, research in this field has largely documented the scale of the problem—poor diet quality, malnutrition risk, and cardiometabolic burden in psychiatric populations—and has evaluated individual nutrition or lifestyle interventions, whereas comparatively few studies have examined how the inpatient meal concept as a whole should be designed [14,17,18]. The catering concept of a psychiatric hospital—the set of overarching goals, dietary forms, meal characteristics, and organisational processes through which patients are fed—is rarely examined as a therapeutic instrument in its own right, and is frequently shaped as much by budgetary and operational constraints, including reimbursement frameworks such as SwissDRG, as by clinical aims. In particular, little is known about how the professionals closest to nutritional care in psychiatry would weigh the competing objectives of an inpatient meal concept, from supporting psychological stability and metabolic health to preserving the pleasure, dignity, and autonomy of eating.

Against this background, the Universitäre Psychiatrische Kliniken (UPK) Basel undertook a re-evaluation of its inpatient catering concept. As a first step, we conducted an online expert survey of nutrition professionals working in psychiatric settings in the German-speaking D-A-CH region (Germany, Austria, and Switzerland) to elicit their views on the goals, patient needs, dietary forms, meal characteristics, organisational aspects, and barriers that should inform a redesigned concept. The aims of the present study were threefold: (1) to identify which overarching goals and patient-specific needs nutrition experts prioritise for an inpatient meal concept; (2) to determine which dietary forms, meal characteristics, and organisational aspects are regarded as central; and (3) to characterise the barriers and success factors relevant to its implementation. In doing so, we sought to reposition the inpatient meal from the domain of catering toward that of the therapeutic concept—treating the provision of food as a clinical means through which a psychiatric hospital can recognise and support its most vulnerable patients.

## 2. Materials and Methods

### 2.1. Study Design and Reporting

We conducted a cross-sectional, anonymous, self-administered online expert survey as part of a service-evaluation project to inform the re-evaluation of the inpatient catering concept at the Universitäre Psychiatrische Kliniken (UPK) Basel. The study is reported in accordance with the Checklist for Reporting Results of Internet E-Surveys (CHERRIES) [19]. The completed CHERRIES checklist is provided in the Supplementary Materials (Supplementary Checklist S1). Reporting of the qualitative component follows the Consolidated Criteria for Reporting Qualitative Research (COREQ). The completed checklist, in which items specific to interviews and focus groups are marked as not applicable, is provided as Supplementary Checklist S2.

### 2.2. Participants and Recruitment

Eligible participants were nutrition professionals with experience in, or professional relevance to, nutritional care in psychiatry—principally dietitians, nutritional scientists, dietetic technicians and assistants, and physicians with a nutrition focus—working predominantly in the German-speaking D-A-CH region (Germany, Austria, and Switzerland). Recruitment was purposive and non-probabilistic: invitations with a link to the survey were disseminated through professional and clinical networks—principally the ESSENzPSYCHE network for nutrition in psychiatry—and participation was voluntary. The respondents’ country of practice was not recorded; given the distribution channels, an estimated maximum of approximately half of the responses is likely to have originated from Switzerland, with the remainder from Germany and possibly Austria. The survey platform recorded 71 accesses to the questionnaire, of which 27 respondents answered the introductory background items and 26 provided data that were retained for analysis, corresponding to a participation rate of approximately 37% among those who accessed the survey. Because the survey was distributed as an open survey through professional channels, rather than to a closed, enumerated panel, the size of the invited population could not be determined, and a response rate relative to it could not be computed, which was in line with the limitations of open web surveys [19]. Only completed questionnaires were retained: every record in the analysed dataset is a complete submission, and no partial data were stored for respondents who discontinued the questionnaire. Of the 71 individuals who accessed the survey, 27 answered the introductory background items; all but one of the respondents who did not complete the survey discontinued at or before the background section and, therefore, provided no demographic information. Demographic characteristics could consequently not be compared between respondents who completed the survey and those who did not; such a comparison would, in any case, have rested on a single non-completing respondent. No formal a priori sample-size calculation was performed, consistent with the exploratory, consensus-oriented aim of the study.

### 2.3. Survey Instrument

The questionnaire was developed by the interdisciplinary project team (nutritional therapy, psychiatry, and food services) on the basis of clinical experience and the literature on nutrition in psychiatry and hospital catering; it was a purpose-designed instrument and had not been formally validated, although item wording and content were reviewed for clarity and relevance within the team. Rather than a separate external pilot, the instrument was refined through iterative review within the interdisciplinary project team for clarity, comprehensibility, and content relevance before deployment. Beyond the background items above (work setting, years of experience, and professional qualification), no further personal or demographic data were collected, consistent with the anonymous design. The instrument comprised three parts: (i) respondent background (work setting, years of experience in nutritional care in psychiatry, and professional qualification); (ii) six matrices of items rated on five-point Likert-type scales, covering the overarching goals of a meal concept (8 items), specific patient needs (8 items), the role of dietary forms (8 items), meal characteristics (9 items), organisational aspects (5 items), and barriers in the current catering system (6 items); and (iii) four open-ended questions on the three most important success factors, recommended best practices, perceived risks of the planned change, and any further comments. Scale anchors were specified for each matrix (1 = not important to 5 = very important for goals, needs, meal characteristics, and organisational aspects; 1 = no role to 5 = important role for dietary forms; and 1 = does not apply at all to 5 = fully applies for barriers). The dietary forms (e.g., Mediterranean, plant-based, anti-inflammatory, low-carbohydrate, protein-rich, and therapeutic diets) were presented to respondents by their established labels without accompanying formal definitions, reflecting the specialist nutrition background of the target sample; the rationale and characteristics of the most highly rated pattern is discussed in Section 4.2. Several matrices included an optional free-text “other” field.

The survey was administered in German using the Unipark platform (Tivian XI GmbH, Cologne, Germany). Items were presented as matrices over several consecutive screens (a background section, the six rating matrices, and the open-ended questions); responses to individual items were not forced, and the field period ran from 16 February to 23 March 2026. The number of screens shown per respondent and the time taken to complete the survey were not recorded in the platform configuration used. The full questionnaire is provided in the Supplementary Materials (Supplementary Questionnaire S1).

### 2.4. Data Preparation

Responses were exported from the survey platform and analysed at the item level. The platform field report lists 27 respondents who answered the introductory background items, of whom 26 submitted a completed questionnaire; these 26 complete submissions constitute the analytic dataset. One of these respondents entered “Betroffene nicht Fachkraft” (“affected person, not a professional”) in the optional free-text field accompanying the work-setting item, while reporting a background in nutritional science (BSc student) under professional qualification. Because the survey was disseminated specifically through professional and clinical networks for nutrition in psychiatry, and because this respondent reported a nutritional-science background, we interpreted this entry as denoting additional first-person experience of mental illness alongside a nutrition-related background rather than as a statement of ineligibility, and the record was therefore retained in the analytic dataset. We regard this as a judgement call and report its consequences: excluding the record would have left the highest- and lowest-rated item in each of the six domains unchanged, with only minor re-orderings among closely adjacent items, and would have altered item means by no more than 0.07 points. All retained records were complete submissions, with none flagged as test entries or duplicates. Platform-specific codes for missing or non-substantive responses and unanswered matrix rows were recoded as missing; no imputation was performed. Because individual items could be left unanswered, the number of valid responses varied across items (*n* = 24–26 of 26 respondents), and analyses were conducted on the available data for each item (pairwise/item-wise). Item non-response was low and did not appear concentrated in particular items: every rated item retained 24–26 valid responses, including items with potentially higher response burden such as digital intake recording, so missingness is unlikely to have materially affected the descriptive results.

### 2.5. Statistical Analysis

Analyses were descriptive. For each item, we computed the mean (*M*), standard deviation (*SD*), median, number of valid responses, and the proportions of respondents endorsing the two highest categories (ratings of 4 or 5; “agreement”), as well as the proportions endorsing the two lowest categories. Recognising the ongoing debate about the measurement level of single Likert-type items, we report measures of central tendency alongside the full response distributions, and we use item means for descriptive comparison and ranking rather than for inferential purposes [20]. Because the six rating matrices were designed to elicit ratings of distinct, individually interpreted priorities rather than to function as reflective, unidimensional scales intended to be summed into composite scores, and because items were analysed individually rather than aggregated, internal-consistency coefficients (e.g., Cronbach’s α) were not computed, as they would not be readily interpretable for an instrument of this kind. Items were ordered by descending mean within each domain, and the full response distributions were displayed as diverging stacked bar charts [21]. Given the exploratory aim and the small, non-probabilistic sample, no inferential hypothesis testing was undertaken. All analyses were performed in R version 4.3.3 [22], and figures were produced with the ggplot2 package version 2 [23].

### 2.6. Analysis of Free-Text Responses

Responses to the four open-ended questions were analysed using reflexive thematic analysis following the six-phase approach of Braun and Clarke [24]: familiarisation with the data; generation of initial codes; construction of candidate themes; review of the themes against the coded extracts and the dataset as a whole; definition and naming of the themes; and reporting. Coding was inductive and semantic: codes were generated from the data rather than from an a priori framework and were kept close to respondents’ own wording rather than interpreted at a latent level, in keeping with the brevity of the responses, which ranged from single words to several sentences. The analysis is accordingly descriptive and codebook-like in orientation, and we make no claim to a depth of interpretation that data of this kind could not support. Because the corpus was fixed and was coded in full, data saturation in the sense of iterative sampling does not apply; all responses were coded, and no themes beyond those present in the complete dataset could be generated.

Initial coding was performed by one analyst (T.L.) in the original German. The resulting codes, candidate themes, and coded extracts were then reviewed by the interdisciplinary co-author team (nutritional therapy, psychiatry, and food services), and disagreements were resolved by discussion and by returning to the raw extracts. Rather than reporting an inter-rater reliability coefficient—which has limited meaning for a small, semantically coded corpus and is not recommended within a reflexive approach—we have prioritised auditability: the complete coding matrix, comprising every response with the codes and themes assigned to it, is provided in Supplementary Table S3, so that readers may trace the derivation of each theme. Theme prevalence is reported as the number of respondents contributing at least one coded extract to a theme; because a respondent could raise a theme under more than one prompt, the number of coded mentions exceeds the number of respondents. Extracts quoted in the manuscript and in the Supplementary Tables were translated from German for reporting, and the original German responses are reproduced verbatim in Table S3.

**Reflexivity and positionality.** The analysis was conducted by members of the project team responsible for the re-evaluation of the inpatient catering concept at UPK Basel: clinicians and nutrition professionals with an existing commitment to strengthening nutritional care in psychiatry and with prior views about the value of a Mediterranean, plant-forward provision. This insider position afforded close familiarity with the clinical and operational context, but it also carried a risk of reading the responses confirmatorily. To mitigate this, codes were held at the semantic level and close to respondents’ wording; coding was reviewed by co-authors from different professional backgrounds; critical, divergent, and inconvenient responses were deliberately retained and reported rather than smoothed away; and the thematic claims were triangulated against the quantitative ratings, which were generated independently of the free-text analysis.

**Divergent and non-evidence-based responses.** One respondent contributed a cluster of claims that lack an evidence base (for example, universal nutrigenetic and “intestinal ecogram” testing, and the assertion that food- and medication-related “intoxication” causes hallucinations, sepsis, or cardiac arrest). These responses were retained in the dataset, coded, reported as a divergent case in Section 3.9, and flagged as such in Table S3, but they were not incorporated into the thematic synthesis.

### 2.7. Ethics

The study surveyed professionals and did not collect patient data or health-related personal data. Under the Swiss Federal Act on Research involving Human Beings (Human Research Act), anonymous surveys of professionals that do not involve health-related personal data fall outside the scope of the Act and do not require approval by a cantonal ethics committee. This was formally confirmed by the responsible cantonal ethics committee (Ethikkommission Nordwest- und Zentralschweiz, EKNZ), which issued a clarification of responsibility (BASEC-ID Req-2026-00906) determining that the project does not fall within the scope of the Human Research Act (Art. 2 para. 1) and therefore does not require its approval. Participation was voluntary and anonymous, and respondents provided informed consent by proceeding with the survey after being informed of its purpose and use.

## 3. Results

We analysed responses from 26 nutrition professionals working in psychiatric settings. As participation in individual matrix items was optional, the number of valid responses varied slightly between items (*n* = 24–26). Throughout, items were rated on five-point scales; we report the mean (*M*), standard deviation (*SD*), and the proportion of respondents selecting the two highest response categories (ratings of 4 or 5), hereafter referred to as agreement.

### 3.1. Sample Characteristics

The largest group of respondents worked in inpatient care (8/26; 31%), followed by mixed (inpatient and outpatient) settings and outpatient care (each 7/26; 27%), and other settings (4/26; 15%); no respondent worked exclusively in a day-clinic setting. The sample was highly experienced: of the 25 respondents with valid data, 13 (52%) reported more than ten years of experience in nutritional care in psychiatry, and only one (4%) reported less than one year. The most common qualification was dietitian (e.g., a member of the Swiss Association of Dietitians [SVDE]; 10/26; 38%), followed by other professional backgrounds (6/26; 23%; e.g., nutritional science, dietetic management, eating-disorder counselling), an MSc in Nutritional Sciences (5/26; 19%), dietetic technician or assistant (3/26; 12%), and physician with a nutrition focus (2/26; 8%). Sample characteristics are summarised in Table 1 and Figure S6.

### 3.2. Overview

**Figure 1:**
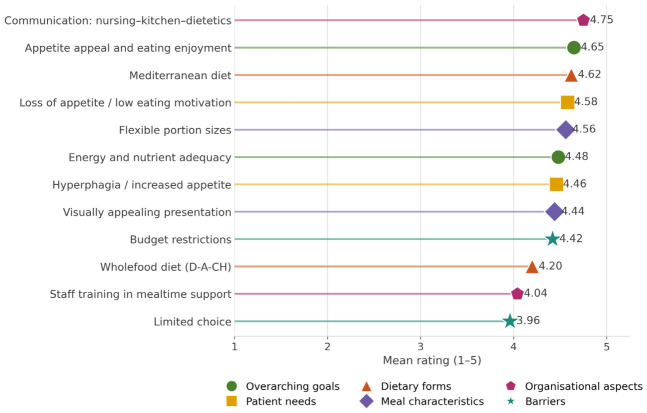
The two highest-rated items within each of the six domains, by mean rating on five-point scales (*n* = 24–26 per item). Domains are distinguished by both colour and marker shape so that they remain legible in greyscale.

Item-level descriptive statistics for all 44 items across the six domains are reported in Table 2, and the full response distributions are provided in Table S1. The two highest-rated items within each domain are shown in Figure 1. Across all domains, the single highest-rated item was interprofessional communication between nursing, kitchen, and dietetics (*M* = 4.75), followed by appetite appeal and eating enjoyment (*M* = 4.65) and the Mediterranean diet (*M* = 4.62).

### 3.3. Overarching Goals of a Meal Concept

**Figure 2:**
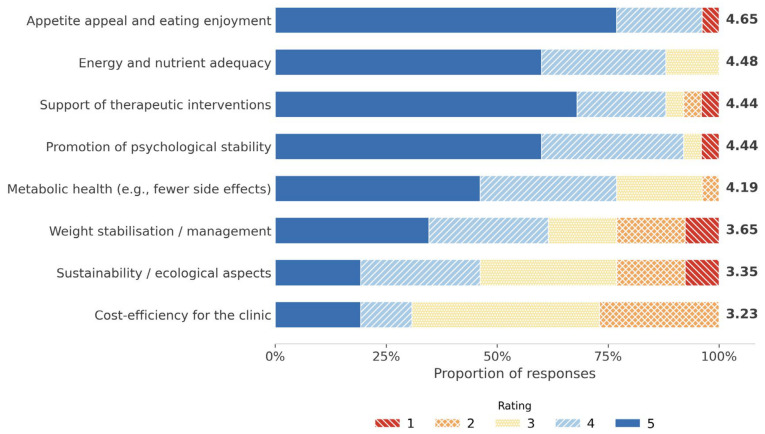
Distribution of importance ratings for the overarching goals of an inpatient meal concept (1 = not important to 5 = very important). Bars show the proportion of responses in each category; mean ratings (*M*) are shown to the right (*n* = 25–26 per item). Rating levels are distinguished by both colour and shading (hatch pattern) so that they remain legible in greyscale.

On the importance scale (1 = not important to 5 = very important), appetite appeal and eating enjoyment received the highest rating (*M* = 4.65, *SD* = 0.85; 96% agreement), ahead of energy and nutrient adequacy (*M* = 4.48), promotion of psychological stability (*M* = 4.44), and support of therapeutic interventions (*M* = 4.44). Metabolic health, framed as the reduction in metabolic side effects, was also rated highly (*M* = 4.19). In contrast, weight stabilisation or management (*M* = 3.65, *SD* = 1.32), sustainability and ecological aspects (*M* = 3.35), and cost-efficiency for the clinic (*M* = 3.23; 31% agreement, 27% disagreement) received the lowest and most dispersed ratings, placing purely economic objectives clearly below patient- and therapy-oriented goals. The full response distribution is shown in Figure 2.

### 3.4. Specific Patient Needs

All eight patient needs were rated as important to address. Loss of appetite or low eating motivation (*M* = 4.58; 88% agreement) and hyperphagia or increased appetite (*M* = 4.46; 92%) were rated highest, indicating that both poles of disordered eating were considered highly relevant. Medication-related concerns—weight gain from psychotropic medication (*M* = 4.44) and metabolic changes from medication (*M* = 4.35)—and eating disorders or restrictive eating (*M* = 4.44) followed closely. Sensory sensitivity or overstimulation (*M* = 3.85), cognitive impairment (*M* = 3.81), and cultural or religious requirements (*M* = 3.50) were rated lower but still above the scale midpoint (Figure S1).

### 3.5. Role of Dietary Forms

On the role scale (1 = no role to 5 = important role), the Mediterranean diet emerged as the clear preferred dietary model (*M* = 4.62; 92% agreement) and showed the lowest dispersion of any item in the survey (*SD* = 0.64). It was followed by a whole-food diet (D-A-CH; *M* = 4.20) and a plant-based diet (*M* = 3.96). An anti-inflammatory orientation (*M* = 3.92) was generally endorsed but with greater dispersion (*SD* = 1.26). Individual special diets (*M* = 3.69), protein-rich diets (*M* = 3.62), and therapeutic diets (*M* = 3.58) occupied the middle range. Low-carb or carbohydrate-reduced options were rated lowest (*M* = 2.65; 23% agreement, 46% disagreement) and were the only dietary form to which respondents predominantly assigned no or a minor role (Figure S2).

### 3.6. Meal Characteristics

Patient-centred and sensory qualities dominated this domain. Flexible portion sizes (*M* = 4.56), a visually appealing presentation (*M* = 4.44), and a clear structure with recognisable foods (*M* = 4.36) were rated most important, followed by high satiety (*M* = 4.32), low sugar and ultra-processing (*M* = 4.24), and simple choice options (*M* = 4.04). Taste palatability was rated somewhat lower (*M* = 3.92). Short waiting times (*M* = 3.44) and, most clearly, beverage variety (*M* = 2.68; 24% agreement) were considered the least important characteristics (Figure S3).

### 3.7. Organisational Aspects

Interprofessional communication between nursing, kitchen, and dietetics was the highest-rated item across the entire survey (*M* = 4.75; 96% agreement). Staff training in mealtime support (*M* = 4.04), the option for individual nutrition plans (*M* = 4.00), and regular nutrition rounds (*M* = 3.88) were also endorsed. Digital recording of food intake was rated lowest and most variably (*M* = 3.04, *SD* = 1.31; 40% agreement), suggesting reservations about its feasibility in routine inpatient care (Figure S4).

### 3.8. Barriers in the Current Catering System

On the applicability scale (1 = does not apply at all to 5 = fully applies), budget restrictions were perceived as the most pronounced barrier (*M* = 4.42; 88% agreement), followed by limited choice (*M* = 3.96) and insufficient quality or freshness (*M* = 3.80). Excessive energy density (*M* = 3.62) and insufficient personalisation (*M* = 3.52) were also endorsed. Difficult collaboration with the kitchen received the lowest rating (*M* = 3.08, *SD* = 1.25); responses were spread across the scale, with the most frequent rating being the midpoint and a minority nonetheless rating it as fully applicable (Figure S5).

### 3.9. Qualitative Findings

**Figure 3:**
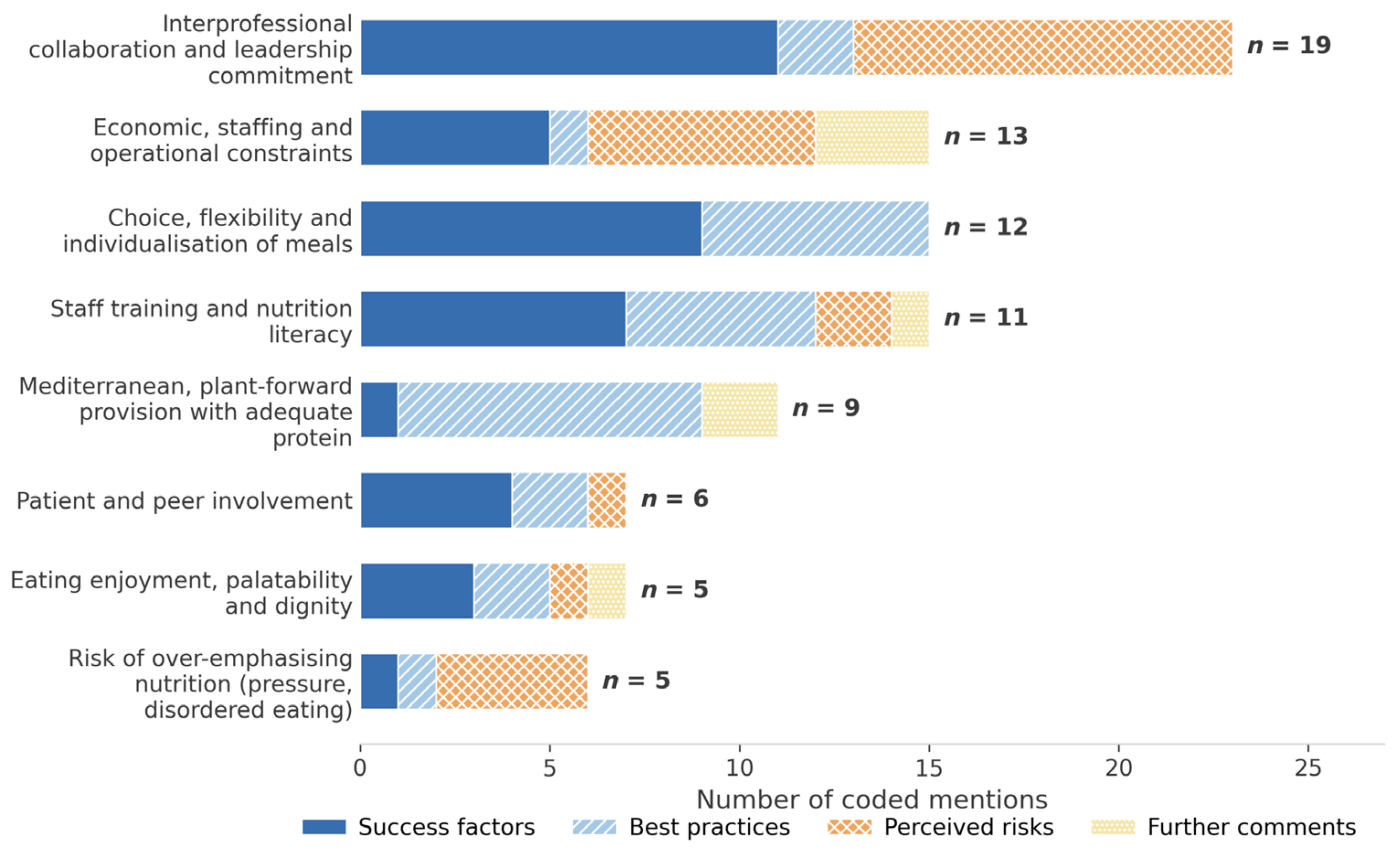
Themes constructed from the free-text responses to the four open-ended questions (22 of the 26 respondents contributed to the thematic synthesis). Bars show the number of coded extracts contributing to each theme, separated by the prompt in which they arose. Because a respondent could raise a theme under more than one prompt, the number of individual respondents contributing to each theme is given at the right (*n*). Prompts are distinguished by both colour and shading (hatch pattern) so that they remain legible in greyscale. The complete coding matrix is provided in Supplementary Table S3.

Free-text responses were provided by 23 of the 26 respondents; those of 22 respondents (85%) entered the thematic synthesis, while those of one respondent were reported separately as a divergent case (Section 2.6). The four open-ended prompts asked for the three most important success factors for the new meal concept, recommended best practices, the main perceived risks of the planned change, and any further comments; responses ranged from single words to several sentences. Eight themes were constructed (Figure 3). The complete coding matrix, with every response and the codes and themes assigned to it, is provided in Table S3, and further illustrative statements are in Table S2.

**Interprofessional collaboration and leadership commitment** were by far the most prevalent theme (19 respondents) and were distinctive in appearing on both sides of the ledger: they were named as the principal success factor and, in their absence, as the principal risk. Respondents described the need to carry the whole system: “Get everyone on board, i.e., sensitise nursing staff, too, to the importance of the meal” (R11, dietitian, >10 years). A change imposed without explanation was expected to provoke resistance—“good communication, with reasons given … otherwise you get pushback” (R03, dietitian, 5–10 years)—and a lack of communication was tersely named as the central risk (R07). Endorsement by clinical and commercial leadership was treated as a precondition (R10, R22), and one respondent framed the danger in terms of professional hierarchy: “The greatest risk is when one of the professions thinks it is more important than the other” (R20). This theme mirrors the quantitative finding that interprofessional communication was the highest-rated item in the entire survey.

**Economic, staffing, and operational constraints** (13 respondents) dominated the risk narrative: “The balance between a variety of diet forms and staff shortages in catering could be a problem” (R04, dietitian, >10 years). Several respondents, however, reframed cost as an argument to be won rather than a fixed ceiling—“Argue financially in the hospital: high quality can also be very economical” (R25, physician, >10 years)—and one reported that a plant-forward conversion had in fact reduced food costs on the ward (R22). Operational feasibility was a recurring caveat: an ordering system must be simple, “otherwise the error rate is high” (R08), and one respondent noted that digital recording of intake was unworkable in a setting with open ward kitchens (R02).

**Choice, flexibility, and individualisation of meals** (12 respondents) was expressed as component- or buffet-based selection with adjustable portions: “A sufficient, varied à la carte or single-component offering that caters for all age groups from 14 to 95” (R02, dietitian, >10 years); “A (limited) choice for patients if they do not like the main menu” (R08, dietitian, >10 years).

**Staff training and nutrition literacy** (11 respondents) were seen as extending across the entire hierarchy rather than residing with dietetics alone: “There is a great deal of ignorance, including among hospital staff: hence training from the director down to the nursing assistant” (R25, physician, >10 years). Practical aids—a diet-forms catalogue, thorough training on the ordering system—were repeatedly requested (R05, R18).

**Mediterranean, plant-forward provision with adequate protein** (9 respondents) was the most frequently recommended best practice: “based on Mediterranean eating, adapted to what is available in Switzerland … enough protein (for good satiety)” (R03, dietitian, 5–10 years); “Mediterranean fare, vegetarian fare without ultra-processed protein sides, full diet with meat-free days” (R04, dietitian, >10 years).

**Patient and peer involvement** (6 respondents) was voiced as the routine incorporation of patient feedback and preferences (R05, R21) and as a stance of attentiveness: “Take them seriously, listen, pay attention to everything—including the asides” (R14).

**Eating enjoyment, palatability, and dignity** (5 respondents) framed the meal as more than the delivery of nutrients: “Enjoyment is addressed through all the senses” (R01, dietitian, 1–3 years). Accessibility was its practical corollary—photograph-based menus, minimal text, and large type for patients whose concentration is impaired (R14).

**Risk of over-emphasising nutrition** (5 respondents) formed an important counterweight and the clearest clinical caution in the dataset. Respondents warned that a nutrition-centric ward culture could itself cause harm: “Fixation on nutrition as the only solution—standards for everyone. May promote eating disorders” (R15, physician, >10 years); “Over-emphasising the importance of dietary details tends to create insecurity” (R16). The latter respondent insisted that “unmonitored freedom and the possibility of enjoyment (including sweets) must be preserved”, while another required dietetic counselling whenever a patient wished to omit several food groups to avoid undersupply (R04).

**Divergent case.** One respondent (R19) advanced a cluster of claims that lack an evidence base, including universal nutrigenetic and “intestinal ecogram” testing and the assertion that food- and medication-related “intoxication” causes hallucinations, sepsis, or cardiac arrest. These responses were coded and are reported here for transparency, but they were not incorporated into the thematic synthesis (Section 2.6; Table S3).

## 4. Discussion

In this expert survey, nutrition professionals working in psychiatric settings prioritised a meal concept built around eating enjoyment and psychological stability rather than economy; rated both poles of disordered eating—appetite loss and hyperphagia—and medication-related metabolic change as highly relevant patient needs; endorsed a Mediterranean, plant-forward dietary model while expressing reservations about low-carbohydrate approaches; and valued flexibility, recognisability, and visual appeal in the meal itself. Strikingly, the single highest-rated item across the entire survey was interprofessional communication between nursing, kitchen, and dietetics, while budget restrictions were seen as the principal barrier. Read together, these priorities describe an inpatient meal that is conceived less as catering and more as a clinical process. The following sections situate these findings within the wider evidence and consider their implications.

### 4.1. Beyond National Dietary Guidelines: Why Psychiatric Inpatients Require Tailored Provision

Public institutions—including hospitals, schools, and care homes—commonly default to the food-based dietary guidelines of their national nutrition societies, such as the German Nutrition Society (DGE) or the Swiss Society for Nutrition (SGE). These guidelines provide a valuable population-level baseline, but they are explicitly designed “for all” and may be insufficient, or even ill-suited, for metabolically atypical subgroups. It has been argued that uniform, population-wide recommendations sit uneasily with a population in which overweight, obesity, prediabetes, and insulin resistance are common and rising, and that such one-size-fits-all guidance may not benefit—and could disadvantage—large metabolic subgroups [25]. Independent of the merits of that particular critique, psychiatric inpatients represent precisely such a subgroup, in concentrated form.

The rationale for special consideration is quantitative. Metabolic disease is markedly more common in severe mental illness (SMI) than in the general population: the risk of diabetes is roughly two to three times higher and that of cardiovascular disease up to four times higher, leading some authors to advocate the term “metabolic psychiatry” [26]. Metabolic syndrome affects approximately one in three people with schizophrenia and related disorders in pooled analyses [27], a figure echoed by recent meta-analytic data from large schizophrenia populations [28]. European position statements likewise document substantially elevated cardiovascular and diabetes risk in this group [13]. Our respondents’ emphasis on metabolic health and on medication-related weight and metabolic change as priority needs mirrors this epidemiology. It follows that psychiatric hospital food provision warrants special attention and deliberate dietary choices rather than the mere adoption of a general national standard—choices that are examined in the two sections that follow, which consider the dietary patterns our respondents most clearly favoured and questioned.

### 4.2. Why the Mediterranean Diet? Evidence and Its Limits

The strong preference our respondents expressed for a Mediterranean pattern is consistent with a substantial—though not uncontested—evidence base linking this diet to mental health. Large syntheses report that higher adherence is associated with a reduced risk of depression and of neurodegenerative diseases, such as Alzheimer’s and Parkinson’s disease [29], and a broad review of more than one hundred observational and interventional studies has described a generally favourable association with mental health, plausibly mediated by anti-inflammatory and antioxidant effects and by beneficial changes in the gut microbiota [3]. At the trial level, a meta-analysis of randomised controlled trials found that Mediterranean-diet interventions produced a moderate reduction in depressive symptoms (standardised mean difference ≈ −0.5) [30]. An eight-week randomised trial reported that a Mediterranean-style lifestyle intervention was non-inferior to psychotherapy for depressive symptoms [31]. Protective associations have also been described in children and adolescents, pointing to a possible preventive role across the life course [32].

The strength of this evidence should nonetheless not be overstated. The same trial meta-analysis rated the certainty of evidence as low, with high heterogeneity and a non-trivial risk of bias, and called for larger, higher-quality studies [30]; observational effect sizes are modest and sensitive to analytic choices, as illustrated by a meta-analysis that initially found no prospective association and whose null result was subsequently attributed to specific methodological decisions, with a corrected re-analysis restoring a significant inverse association [33]. The Mediterranean diet is therefore best understood as a well-tolerated, broadly health-promoting pattern with reasonable—if still maturing—support for mental health, rather than as a proven treatment. Its suitability as the basis for an inpatient concept rests not only on this evidence but also on its established cardiometabolic benefits, its flexibility and palatability, and its compatibility with the enjoyment- and dignity-centred goals our experts prioritised. It is worth adding that the environmental co-benefits often attributed to such patterns are not automatic and may need to be designed for explicitly [34]—a nuance relevant to the relatively modest weight our respondents placed on sustainability as a goal.

### 4.3. Low-Carbohydrate Diets in Perspective: From Dietary Pattern to Metabolic Therapy

That experts assigned low-carbohydrate options the lowest rating of any dietary form deserves careful interpretation rather than dismissal. As a general catering default, a low-carbohydrate orientation is indeed difficult to justify: it sits awkwardly beside the Mediterranean, plant-forward pattern that attracted the strongest support, and it can become monotonous and hard to sustain when scaled to a whole ward. A blanket low-carbohydrate menu for a heterogeneous inpatient population is, on present evidence, not advisable, and the low rating is best read as appropriate caution about a population-level default.

This caution should not, however, be interpreted as a verdict against carbohydrate restriction in all its forms. An important distinction must be drawn: a moderately reduced-carbohydrate diet represents a dietary pattern, whereas a ketogenic diet pursued for clinical purposes—ketogenic metabolic therapy—is a fundamentally different intervention. It is a structured therapeutic approach with pronounced and measurable metabolic effects, and emerging clinical evidence suggests that it may benefit carefully selected patients with specific psychiatric conditions [35]. Precisely for this reason, it should not be regarded as routine dietary provision. Expert consensus emphasises that ketogenic metabolic therapy requires careful candidate selection, screening for contraindications, monitoring of electrolytes and micronutrients, proactive adjustment of psychotropic and other medications when appropriate, and structured clinical supervision, reflecting the principle that such interventions are not suitable for everyone [36]. Ketogenic metabolic therapy therefore belongs within the framework of individualised treatment, delivered to selected patients after informed consent and under appropriate supervision [35], rather than as a general dietary service. The low rating assigned to low-carbohydrate catering and the carefully governed clinical use of ketogenic therapy in selected patients are therefore not contradictory, but represent two distinct levels within the same framework: a sensible population-level dietary default on the one hand, and a personalised metabolic therapy on the other.

### 4.4. Designing for the Appetite Spectrum: Medication, Hunger, and Satiety

That experts rated both appetite loss and increased appetite among the most important patient needs reflects a genuine double burden in psychiatric care. Hunger and satiety are physiologically vulnerable processes that are readily dysregulated [37], and psychotropic medication acts directly on the systems that govern food intake: the appetite-modulating effects of psychiatric drugs have been recognised for decades [38], and antipsychotics in particular are well-established contributors to weight gain and obesity in SMI [39]. At the opposite pole, poor oral intake in psychiatric patients has identifiable, clinically meaningful explanations that range from depressive anorexia to cognitive and psychotic symptoms [40]. The nutritional dimension of psychiatric disorder is thus neither new nor peripheral [41]. These mechanisms make sense of our experts’ emphasis on flexible portion sizes, recognisable foods, adequate satiety, and visually appealing presentation: design features that allow a single catering system to accommodate patients at both ends of the appetite spectrum without resorting to restriction.

### 4.5. The Overlooked Risks: Malnutrition and Food Insecurity

Discussion of nutrition in SMI is often dominated by obesity, yet undernutrition is a substantial and under-recognised risk as well. A considerable proportion of long-term inpatients with schizophrenia are at risk of malnutrition [42]; nutritional risk predicts poorer clinical prognosis in chronic schizophrenia [43], and malnutrition has been associated with longer length of stay on acute psychiatric wards [6]—a direct parallel to the general-hospital evidence that treating nutritional risk improves outcomes [8]. The problem extends beyond the ward: malnutrition has identifiable determinants among community-dwelling schizophrenia patients [44], and food insecurity is disproportionately common in people with SMI [45,46]. Together, these findings support the view that nutritional therapy is a legitimate clinical target in mental-health care [47] and reframe the inpatient meal as a safeguard against both metabolic harm and undernutrition.

### 4.6. From Provision to Process: Communication, Choice, and Patient Involvement

Perhaps the most policy-relevant finding is that the highest-rated item overall was not a food or a diet but a process—interprofessional communication between nursing, kitchen, and dietetics—reinforced in the free-text responses by themes of collaboration, leadership endorsement, individualisation, and patient involvement. This aligns with evidence that structured nutrition and dietary interventions can improve metabolic risk factors in SMI [14] and that multimodal, individualised weight-management programmes are feasible in this population [48]. It also reflects a demand side that is easily overlooked: patients with mental disorders themselves express a need for nutritional counselling, particularly those with dietary problems and lower quality of life [18]. Because obesity and adverse nutrition behaviours occur across diagnostic and cultural contexts [49], and because Swiss psychiatric patients specifically show poorer nutritional status, disordered eating patterns, and concrete barriers to healthy eating [15,16], a choice-based, individualised model—supported by interprofessional coordination and staff training—appears better suited to this setting than a uniform menu. The case for treating the meal as part of the clinical process is reinforced by evidence that diet is not incidental to psychiatric illness even at the level of biology: dietary intake correlates with polygenic risk for schizophrenia and bipolar disorder [50], and dietary patterns have been linked to outcomes as serious as suicide [51].

### 4.7. Implications for the Inpatient Meal Concept

Several practical implications follow for the redesign of an inpatient catering concept. First, the nutritional baseline should be a Mediterranean, plant-forward but protein-adequate pattern rather than an unmodified national guideline or a restrictive therapeutic diet. Second, the system should be flexible and choice-based—through component selection, buffet, or plate models with guided portioning—so that it can serve both diminished and excessive appetites. Third, meals should be recognisable, visually appealing, and lower in sugar and ultra-processing, features that support intake without coercion. Fourth, the meal concept should be embedded in interprofessional processes, including routine nutritional risk screening, staff training, and clear communication channels. Finally, although budget was identified as the principal barrier, the general-hospital evidence that nutritional support reduces complications and mortality [8] suggests that high-quality provision is better framed as a clinical investment than as a cost. Realising this vision is consistent with the broader agenda of nutritional psychiatry, which calls for nutrition to be integrated into routine mental-health care [52].

### 4.8. Strengths and Limitations

The principal strength of this study is its access to an experienced, purposively selected group of nutrition professionals and its combination of quantitative ratings with qualitative free-text analysis; to our knowledge, it is among the first studies to elicit expert priorities specifically for an inpatient psychiatric catering concept. Several limitations, however, must temper interpretation. The sample was small, self-selected, and non-probabilistic, and was drawn from the German-speaking D-A-CH region (Germany, Austria, and Switzerland). Because respondents’ country of practice was not recorded, the precise national composition of the sample is unknown, which further limits generalisability. Future studies should also record respondents’ country of practice, since the balance of Swiss versus German and Austrian participants may shape perceptions of budgetary constraints—for example, through country-specific reimbursement systems such as SwissDRG—and could be examined as a moderator of meal-concept priorities. As an open online survey distributed through professional networks, it is subject to coverage and self-selection bias, and the size of the invited population—and hence a response rate—could not be determined; these are recognised inherent limitations of the online survey method [53,54], even though such surveys also offer well-documented advantages in reach and efficiency [55,56]. The instrument was purpose-designed and not formally validated; the analysis was descriptive and exploratory, without inferential testing; and the initial thematic analysis was conducted by a single coder, though theme refinement was discussed among the co-authors. Ratings reflect professional opinion rather than patient-level outcomes. Findings should therefore be read as hypothesis-generating input to a local service redesign rather than as generalisable estimates.

## 5. Conclusions

Because people with SMI carry a disproportionate burden of obesity, metabolic syndrome, malnutrition risk, and food insecurity, and because their appetite and metabolism are shaped by both illness and its treatment, the routine adoption of a general national dietary standard is unlikely to meet their needs. Nutrition professionals broadly converged on a coherent vision for inpatient psychiatric catering: genuinely patient-centred, Mediterranean and plant-forward, flexible and choice-based, and—above all—delivered through strong interprofessional processes. Treating the inpatient meal as a therapeutic component of care—rather than as catering—offers a concrete means by which a psychiatric hospital can recognise and support its most vulnerable patients.

## Figures and Tables

**Table 1:** Characteristics of the surveyed nutrition professionals (*n* = 26). Values are counts (*n*) and percentages of valid responses.

Characteristic	*n*	%
Work setting		
Inpatient	8	31
Day-clinic	0	0
Outpatient	7	27
Mixed setting	7	27
Other	4	15
Experience (years)		
<1 year	1	4
1–3 years	5	20
3–5 years	3	12
5–10 years	3	12
10+ years	13	52
Qualification		
Dietitian (e.g., SVDE)	10	38
Dietetic technician/assistant	3	12
MSc Nutritional Sciences	5	19
Physician, nutrition focus	2	8
Other	6	23

SVDE, Swiss Association of Dietitians (Schweizerischer Verband der Ernährungsberater/innen). Percentages for work setting and qualification are based on all 26 respondents; percentages for experience are based on the 25 respondents with valid data (one missing). Percentages are rounded to the nearest integer and may not sum to exactly 100.

**Table 2:** Item-level descriptive statistics for all 44 items across the six domains.

Item	*n*	*M*	*SD*	*Mdn*	IQR (Q1–Q3)	Agreement (4–5, %)	Disagreement (1–2, %)
Overarching goals							
Appetite appeal and eating enjoyment	26	4.65	0.85	5	5–5	96	4
Energy and nutrient adequacy	25	4.48	0.71	5	4–5	88	0
Promotion of psychological stability	25	4.44	0.92	5	4–5	92	4
Support of therapeutic interventions	25	4.44	1.04	5	4–5	88	8
Metabolic health (e.g., fewer side effects)	26	4.19	0.90	4	4–5	77	4
Weight stabilisation/management	26	3.65	1.32	4	3–5	62	23
Sustainability/ecological aspects	26	3.35	1.20	3	3–4	46	23
Cost-efficiency for the clinic	26	3.23	1.07	3	2.2–4	31	27
Patient needs							
Loss of appetite/low eating motivation	26	4.58	0.95	5	5–5	88	4
Hyperphagia/increased appetite	26	4.46	0.99	5	4–5	92	8
Weight gain from psychotropic medication	25	4.44	0.96	5	4–5	84	8
Eating disorders/restrictive eating	25	4.44	0.96	5	4–5	88	4
Metabolic changes from medication	26	4.35	0.94	5	4–5	85	8
Sensory sensitivity/overstimulation	26	3.85	0.92	4	3.2–4	73	12
Cognitive impairment	26	3.81	0.85	4	3–4	62	4
Cultural or religious requirements	26	3.50	1.21	4	2.2–4	58	27
Dietary forms							
Mediterranean diet	26	4.62	0.64	5	4–5	92	0
Whole-food diet (D-A-CH)	25	4.20	0.96	4	4–5	84	4
Plant-based diet	26	3.96	1.00	4	3–5	69	4
Anti-inflammatory diet	26	3.92	1.26	4.5	3–5	62	15
Individual special diets	26	3.69	0.97	3.5	3–4.8	50	8
Protein-rich diet	26	3.62	0.98	4	3–4	58	15
Therapeutic diets (diabetes, lipids)	26	3.58	1.21	4	3–4	62	19
Low-carb/carbohydrate-reduced	26	2.65	1.20	3	2–3	23	46
Meal characteristics							
Flexible portion sizes	25	4.56	0.82	5	4–5	88	4
Visually appealing presentation	25	4.44	0.65	5	4–5	92	0
Clear structure/recognisable foods	25	4.36	0.76	4	4–5	92	4
High satiety	25	4.32	0.90	5	4–5	80	4
Low sugar and ultra-processing	25	4.24	0.88	4	4–5	80	4
Simple choice options	25	4.04	0.79	4	4–5	80	4
High taste palatability	24	3.92	0.88	4	3–5	67	4
Short waiting times	25	3.44	0.92	3	3–4	48	16
Beverage variety	25	2.68	1.14	3	2–3	24	40
Organisational aspects							
Communication: nursing–kitchen–dietetics	24	4.75	0.68	5	5–5	96	4
Staff training in mealtime support	25	4.04	0.98	4	3–5	72	8
Option for individual nutrition plans	25	4.00	1.08	4	4–5	76	16
Regular nutrition rounds	25	3.88	1.20	4	3–5	72	12
Digital recording of food intake	25	3.04	1.31	3	2–4	40	28
Barriers							
Budget restrictions	24	4.42	0.93	5	4–5	88	8
Limited choice	24	3.96	1.16	4	4–5	79	17
Insufficient quality/freshness	25	3.80	1.32	4	3–5	64	24
Excessive energy density	24	3.62	1.10	4	3–4	63	17
Insufficient personalisation	25	3.52	1.16	4	3–4	60	24
Difficult collaboration with the kitchen	24	3.08	1.25	3	2–4	29	33

*M*, mean; *SD*, standard deviation; *Mdn*, median; IQR, interquartile range (first to third quartiles); *n*, valid responses. Agreement = percentage of respondents selecting the two highest response categories (4–5); Disagreement = the two lowest (1–2). Items are ordered by domain and by descending mean. Response scales: for overarching goals, patient needs, meal characteristics, and organisational aspects, 1 = not important to 5 = very important; for dietary forms, 1 = no role to 5 = important role; for barriers, 1 = does not apply at all to 5 = fully applies. Percentages are rounded to the nearest integer.

## Data Availability

The aggregated data presented in this study are available within the article and its Supplementary Materials. The de-identified item-level dataset is available from the corresponding author upon reasonable request; it is not publicly available because it forms part of an internal service-evaluation project.

## Supplementary Materials

The following supporting information can be downloaded at https://www.mdpi.com/article/10.3390/nu18152448/s1, Table S1: Full response distributions (percentage of responses in each category, 1–5) for all 44 items across the six domains; for each item the categories run from 1 (lowest) to 5 (highest) on the respective scale; Figure S1: Distribution of importance ratings for specific patient needs (1 = not important to 5 = very important; n = 25–26 per item); Figure S2: Distribution of ratings for the role of dietary forms in the new concept (1 = no role to 5 = important role; n = 25–26 per item); Figure S3: Distribution of importance ratings for meal characteristics (1 = not important to 5 = very important; n = 24–25 per item); Figure S4: Distribution of importance ratings for organisational aspects (1 = not important to 5 = very important; n = 24–25 per item); Figure S5: Distribution of ratings for barriers in the current catering system (1 = does not apply at all to 5 = fully applies; n = 24–25 per item); Figure S6: Sample characteristics: work setting, professional experience, and qualification (n = 26; valid n = 25 for experience); Table S2: Representative free-text statements by theme, translated and synthesised from the German open-ended responses, illustrating expert priorities, recommended practices, and perceived risks; Table S3: Complete qualitative coding matrix: every free-text response to the four open-ended prompts, with the codes and themes assigned to it; Supplementary Questionnaire S1: Survey instrument; Supplementary Checklist S1: Completed CHERRIES (Checklist for Reporting Results of Internet E-Surveys) [19]; Supplementary Checklist S2: Completed COREQ (Consolidated Criteria for Reporting Qualitative Research) checklist for the qualitative component [57].

## Abbreviations

CHERRIES, Checklist for Reporting Results of Internet E-Surveys; COREQ, Consolidated Criteria for Reporting Qualitative Research; D-A-CH, Germany (D), Austria (A) and Switzerland (CH); DGE, German Nutrition Society; *M*, mean; SD, standard deviation; SMI, severe mental illness; SVDE, Swiss Association of Dietitians; UPK, Universitäre Psychiatrische Kliniken Basel.
