# Supplementary material for: From Catering to Clinical Care: An Expert Survey on Designing the Inpatient Psychiatric Meal Concept
Source: Nutrients. 2026 Jul 27;18(15):2448. doi: 10.3390/nu18152448 (PMC13468639; doi:10.3390/nu18152448)
Supplement: Supplementary file 1 [file nutrients-18-02448-s001.zip › nutrients-4407616-supplementary.pdf]

Supplementary Materials

From Catering to Clinical Care: An Expert Survey on Designing the Inpatient Psychiatric Meal Concept

Timur Liwinski[1,2,†,*], Sandra Nussbaum[1,†], Lukas Imfeld[1], Ulf Wein[1], Undine Lang[1,2]

[1] University Psychiatric Clinics Basel, Clinic for Adults, University of Basel, Basel, Switzerland.
[2] University of Basel, Basel, Switzerland.

* Correspondence: Timur Liwinski; timur.liwinski@upk.ch

† These authors contributed equally to this work and share first authorship.

This document contains the supplementary tables and figures referenced in the main text. Table S1 provides the complete item-level response distributions, Figures S1–S5 present the full response distributions for the survey domains not shown in the main text, Figure S6 summarises the sample composition, and Table S2 lists representative free-text statements. The full survey instrument is reproduced in both the original German and an English translation (Supplementary Questionnaire S1), Table S3 gives the complete qualitative coding matrix, and the completed CHERRIES and COREQ reporting checklists (Supplementary Checklists S1 and S2) are appended at the end. All ratings were made on five-point scales; bars in the figures show the proportion of responses in each category, and mean ratings (M) are shown to the right of each bar.

Table S1. Full response distributions (percentage of responses in each category, 1–5) for all 44 items across the six domains; for each item the categories run from 1 (lowest) to 5 (highest) on the respective scale. n denotes the number of valid responses per item.

Item	1	2	3	4	5	n
**Overarching goals**						
Promotion of psychological stability	4	0	4	32	60	25
Energy and nutrient adequacy	0	0	12	28	60	25
Appetite appeal and eating enjoyment	4	0	0	19	77	26
Metabolic health (e.g., fewer side effects)	0	4	19	31	46	26
Weight stabilisation / management	8	15	15	27	35	26
Support of therapeutic interventions	4	4	4	20	68	25
Sustainability / ecological aspects	8	15	31	27	19	26
Cost-efficiency for the clinic	0	27	42	12	19	26
**Patient needs**						
Loss of appetite / low eating motivation	4	0	8	12	77	26
Hyperphagia / increased appetite	4	4	0	27	65	26
Weight gain from psychotropic medication	0	8	8	16	68	25
Metabolic changes from medication	0	8	8	27	58	26
Eating disorders / restrictive eating	4	0	8	24	64	25
Cognitive impairment	0	4	35	38	23	26
Sensory sensitivity / overstimulation	0	12	15	50	23	26
Cultural or religious requirements	4	23	15	35	23	26
**Dietary forms**						
Mediterranean diet	0	0	8	23	69	26
Wholefood diet (D-A-CH)	4	0	12	40	44	25
Therapeutic diets (diabetes, lipids)	8	12	19	38	23	26
Protein-rich diet	0	15	27	38	19	26
Plant-based diet	4	0	27	35	35	26
Low-carb / carbohydrate-reduced	19	27	31	15	8	26
Anti-inflammatory diet	4	12	23	12	50	26
Individual special diets	0	8	42	23	27	26
**Meal characteristics**						

Item	1	2	3	4	5	n
High satiety	0	4	16	24	56	25
Clear structure / recognisable foods	0	4	4	44	48	25
Short waiting times	0	16	36	36	12	25
Simple choice options	0	4	16	52	28	25
High taste palatability	0	4	29	38	29	24
Visually appealing presentation	0	0	8	40	52	25
Flexible portion sizes	0	4	8	16	72	25
Beverage variety	20	20	36	20	4	25
Low sugar and ultra-processing	0	4	16	32	48	25
**Organisational aspects**						
Communication: nursing–kitchen–dietetics	0	4	0	12	83	24
Regular nutrition rounds	8	4	16	36	36	25
Staff training in mealtime support	0	8	20	32	40	25
Digital recording of food intake	20	8	32	28	12	25
Option for individual nutrition plans	0	16	8	36	40	25
**Barriers**						
Limited choice	4	12	4	42	38	24
Insufficient quality / freshness	4	20	12	20	44	25
Excessive energy density	4	12	21	42	21	24
Insufficient personalisation	4	20	16	40	20	25
Budget restrictions	0	8	4	25	62	24
Difficult collaboration with the kitchen	8	25	38	8	21	24

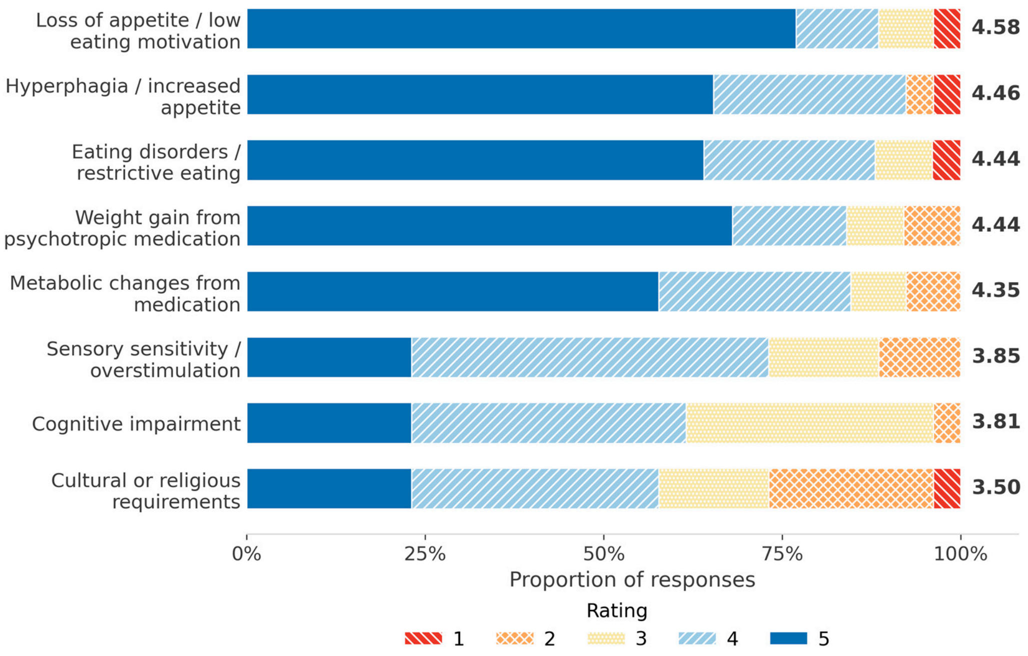

Figure S1. Distribution of importance ratings for specific patient needs (1 = not important to 5 = very important; n = 25–26 per item).

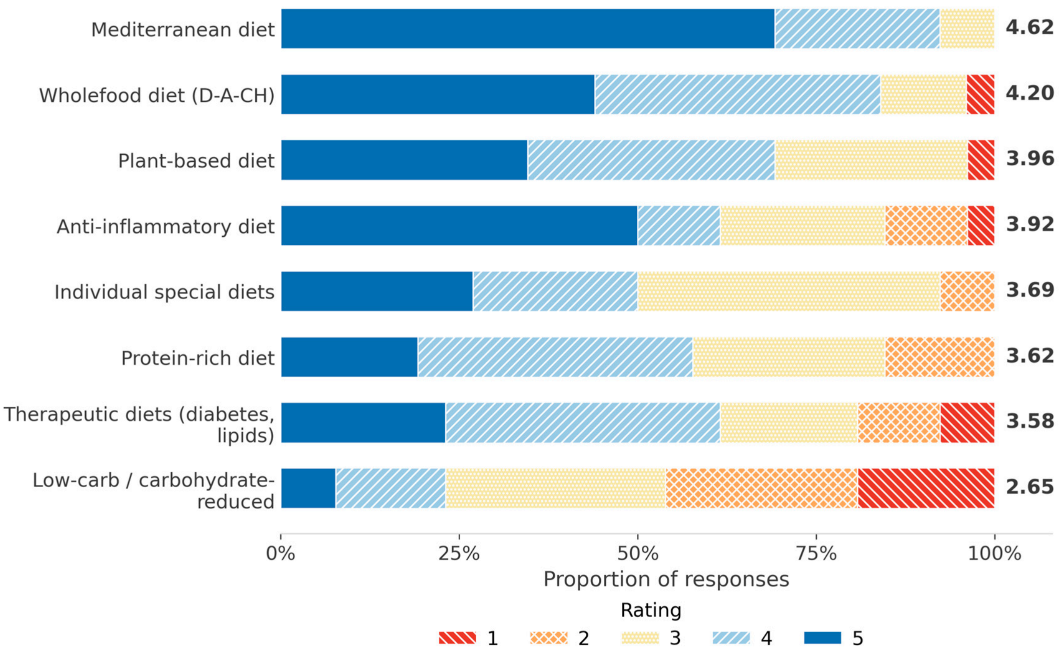

Figure S2. Distribution of ratings for the role of dietary forms in the new concept (1 = no role to 5 = important role; n = 25–26 per item).

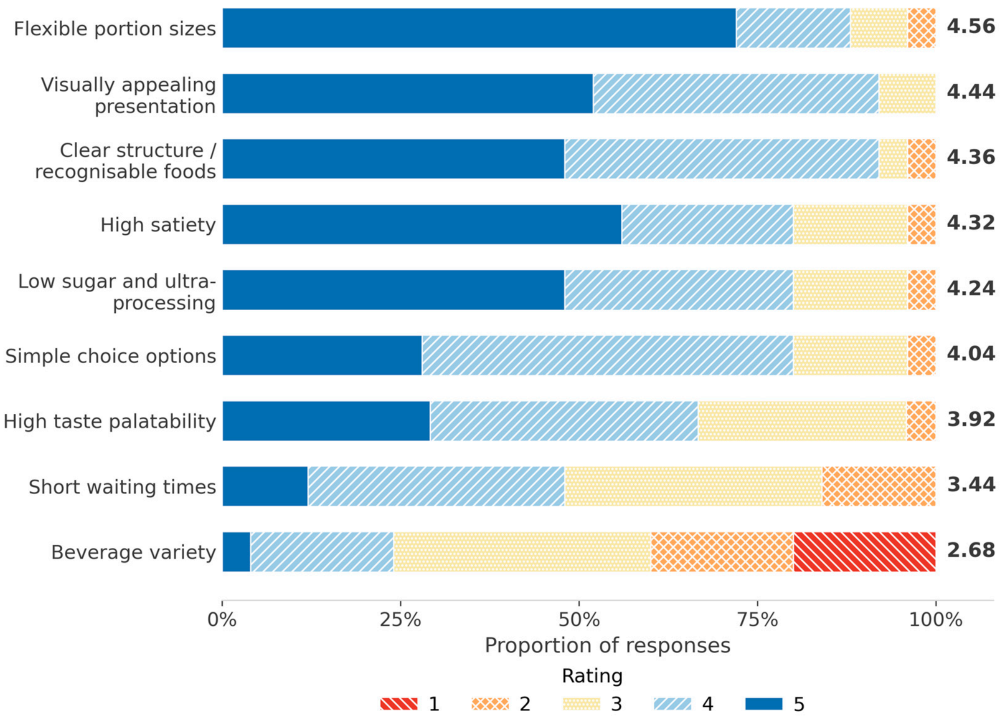

Figure S3. Distribution of importance ratings for meal characteristics (1 = not important to 5 = very important; n = 24–25 per item).

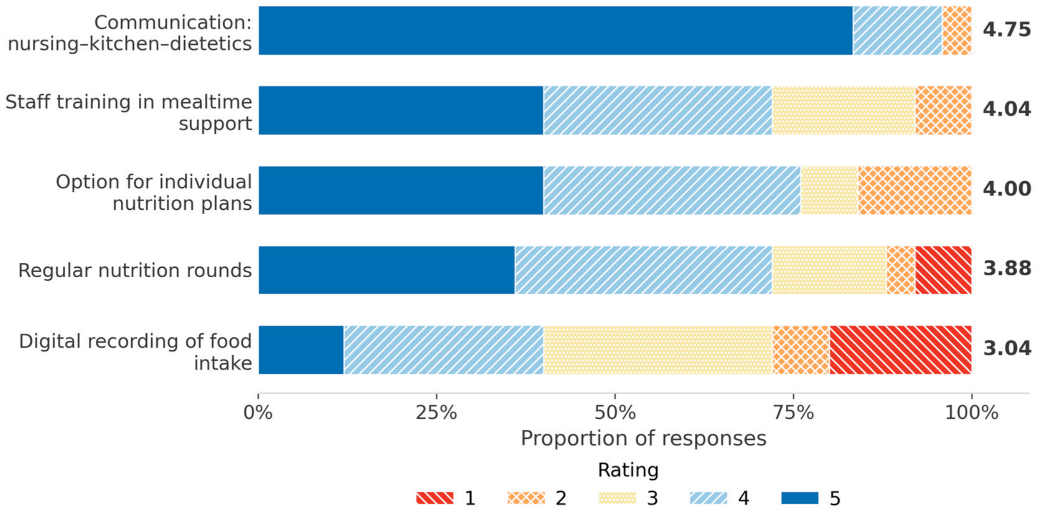

Figure S4. Distribution of importance ratings for organisational aspects (1 = not important to 5 = very important; n = 24–25 per item).

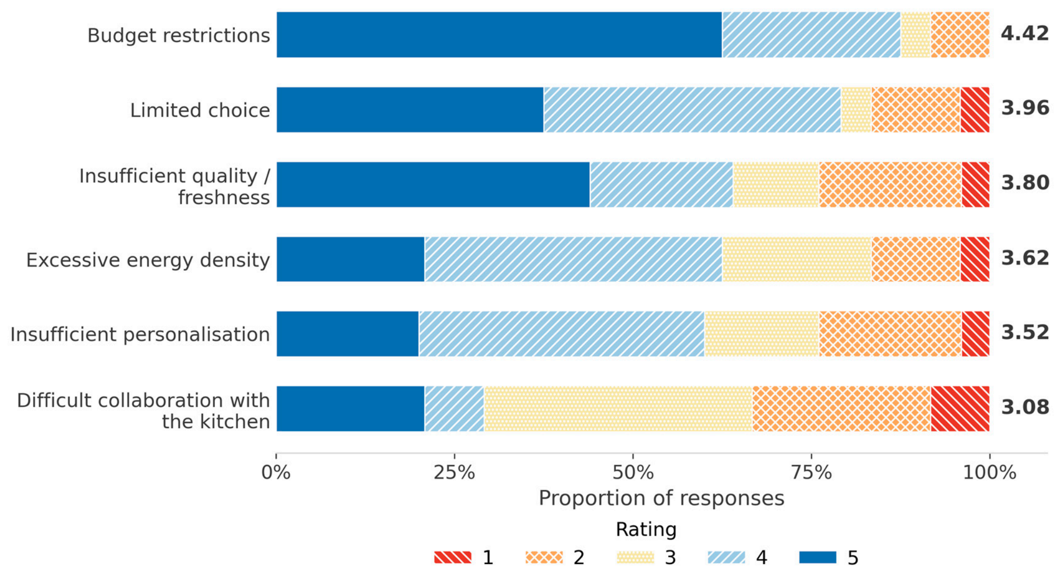

Figure S5. Distribution of ratings for barriers in the current catering system (1 = does not apply at all to 5 = fully applies; n = 24–25 per item).

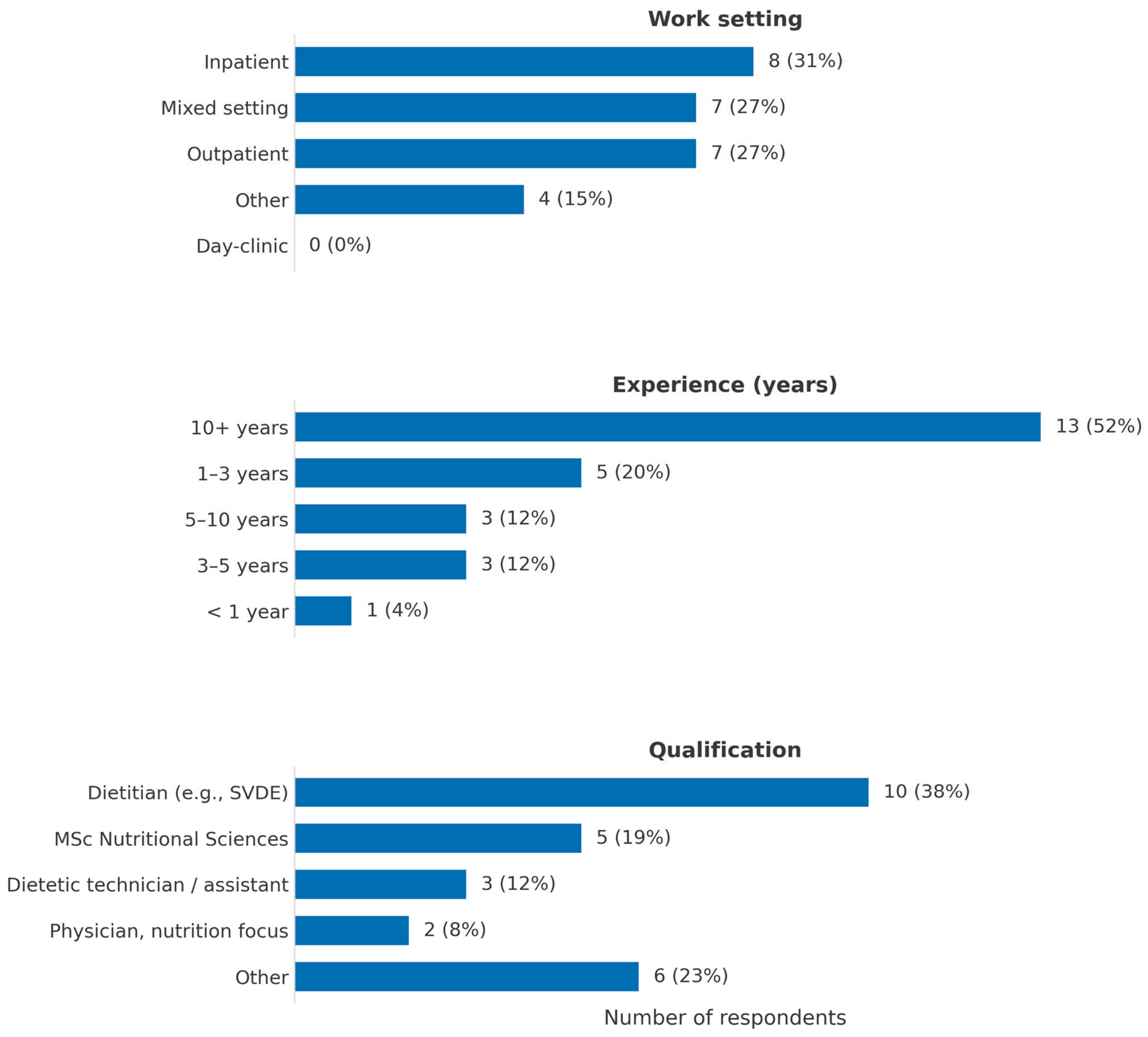

Figure S6. Sample characteristics: work setting, professional experience, and qualification (n = 26; valid n = 25 for experience).

Table S2. Representative free-text statements by theme, translated and synthesised from the German open-ended responses, illustrating expert priorities, recommended practices, and perceived risks.

Theme	Representative statements
**Success factors**	
Interprofessional collaboration & leadership	Success depends on getting nursing, the kitchen, and dietetics to pull together, with clear backing from management; without shared ownership the concept will not be sustained.
Choice & individualisation	Patients should be able to choose from components or a buffet so that meals can be adapted to individual needs and preferences rather than imposed uniformly.
Enjoyment & dignity	Eating should remain a pleasant, dignified part of the day; meals should not be reduced to rules and restrictions.
Communication, training & simple ordering	Clear information flow, trained staff at mealtimes, and a simple, low-barrier ordering system were seen as practical prerequisites.
**Recommended best practices**	
Mediterranean, plant-forward base with adequate protein	A Mediterranean, largely plant-based orientation with sufficient protein for satiety was repeatedly recommended as the everyday standard.
Buffet or plate model with guided portioning	Buffet or plate-model service with staff-guided self-portioning helps accommodate both small and large appetites.
Picture-based, low-text menus	Menus using pictures and simple language support patients with cognitive or language difficulties.
Patient and peer involvement; familiar dishes	Involving patients (and peers) and offering familiar dishes in a nutritionally adapted form improves acceptance.
**Perceived risks**	
Variety versus budget and staffing	The main tension was providing appealing variety and quality within tight budget and staffing limits.
Staff resistance and workload	New ordering systems and changed routines may meet resistance and add to staff workload if poorly supported.
Over-emphasis on nutrition	An excessive focus on nutrition could create pressure and prove counterproductive, particularly for patients with eating disorders.
Under-provision	Cutting provision too far risks leaving patients inadequately nourished.

Supplementary Questionnaire S1. Survey instrument

The questionnaire was administered online in German via Unipark. Part A reproduces the original German instrument; Part B is an English translation. Within each rating matrix, every item was rated on the five-point scale indicated; free-text fields are marked accordingly. The instrument is reconstructed from the survey field report.

A. Originalversion (Deutsch)

Teil 1 – Hintergrund

1. In welchem Setting arbeiten Sie überwiegend?

- Stationär
- Tagesklinisch
- Ambulant
- Mischform
- Sonstiges (bitte spezifizieren): _______________

2. Wie viele Jahre Berufserfahrung haben Sie in der Ernährungsberatung in der Psychiatrie?

- < 1 Jahr
- 1–3 Jahre
- 3–5 Jahre
- 5–10 Jahre
- 10+ Jahre

3. Welche Qualifikation trifft am besten auf Sie zu?

- Ernährungsberater:in (z. B. SVDE)
- Diätolog:in / Diätassistent:in
- MSc Ernährungswissenschaften
- Arzt/Ärztin mit Ernährungsschwerpunkt
- Sonstiges (bitte spezifizieren): _______________

Teil 2 – Bewertungen

4. Bei den folgenden Fragen geht es um Qualitäten eines Verpflegungskonzeptes. Wie wichtig sind Ihnen die folgenden übergeordneten Ziele eines Verpflegungskonzepts in der Psychiatrie?

(1 = unwichtig ... 5 = sehr wichtig)

Item	1	2	3	4	5
Förderung der psychischen Stabilität					
Energie- und Nährstoffadäquanz					
Essanreiz und Essfreude					
Stoffwechselgesundheit (z. B. Reduktion metabolischer Nebenwirkungen)					
Gewichtsstabilisierung / Gewichtsmanagement					
Unterstützung therapeutischer Interventionen					

Nachhaltigkeit / ökologische Aspekte					
Wirtschaftlichkeit für die Klinik					

5. Welche spezifischen Bedürfnisse sollten bei psychiatrischen Patientinnen und Patienten besonders berücksichtigt werden?

(1 = unwichtig ... 5 = sehr wichtig)

Item	1	2	3	4	5
Appetitlosigkeit / mangelnde Essmotivation					
Hyperphagie / gesteigerter Appetit					
Gewichtszunahme durch Psychopharmaka					
Stoffwechselveränderungen durch Medikamente					
Essstörungen / restriktives Essverhalten					
Kognitive Einschränkungen					
Reizoffenheit / sensorische Überforderung					
Kulturelle oder religiöse Anforderungen					
Sonstige (bitte spezifizieren): _______________					

6. Welche Rolle sollten folgende Kostformen im neuen Konzept spielen?

(1 = keine Rolle ... 5 = wichtige Rolle)

Item	1	2	3	4	5
Mediterrane Kost					
Vollwertkost (D-A-CH)					
Therapeutische Diäten (Diabetes, Lipidstoffwechsel, etc.)					
Proteinbetonte Kost					
Pflanzlich betonte Kost					
Low-Carb / Kohlenhydrat-reduzierte Optionen					
Anti-entzündliche Kost					
Individuelle Sonderkostformen					

7. Bei den folgenden Fragen möchten wir Ihre Meinung zur Gestaltung der Speise und der Prozesse erfahren. Welche Merkmale einer Mahlzeit sind für psychiatrische Patientinnen und Patienten besonders wichtig?

(1 = unwichtig ... 5 = sehr wichtig)

Item	1	2	3	4	5
Hohe Sättigung					
Klare Struktur / gut erkennbare Lebensmittel					
Kurze Wartezeiten					
Einfache Auswahlmöglichkeiten					
Hohe Geschmackspalatabilität					
Visuell ansprechende Präsentation					
Flexibilität bei Portionsgrößen					

Getränkevielfalt					
Niedriger Zucker- und Ultra-Prozessierungsgrad					

8. Wie wichtig sind folgende organisatorische Aspekte?

(1 = unwichtig … 5 = sehr wichtig)

Item	1	2	3	4	5
Kommunikation zwischen Pflege, Küche und Ernährungsberatung					
Regelmässige Ernährungsvisiten					
Schulungen der Pflege zu Essbegleitung					
Digitale Erfassung von Nahrungsaufnahme					
Möglichkeit für individuelle Ernährungspläne					

9. Welche Barrieren bestehen häufig im derzeitigen Verpflegungssystem?

(1 = trifft gar nicht zu … 5 = trifft vollkommen zu)

Item	1	2	3	4	5
Limitierte Auswahl					
Ungenügende Qualität / Frische					
Zu hohe Energiedichte					
Unzureichende Personalisierung					
Budgetrestriktionen					
Schwierige Zusammenarbeit mit der Küche					
Sonstiges (bitte spezifizieren): ______________					

Teil 3 – Offene Fragen

10. Welche drei Aspekte sind aus Ihrer Sicht am wichtigsten, damit ein neues Verpflegungskonzept in der Psychiatrie erfolgreich ist?

(Offene Freitextangabe.)

11. Welche Konzepte oder Best Practices aus Ihrer Erfahrung sollten unbedingt integriert werden?

(Offene Freitextangabe.)

12. Gibt es aus Ihrer Sicht Risiken, die bei der Umstellung unbedingt bedacht werden müssen?

(Offene Freitextangabe.)

13. Haben Sie weitere Anmerkungen oder Empfehlungen?

(Offene Freitextangabe.)

B. English translation

Part 1 – Background

1. In which setting do you predominantly work?

- Inpatient
- Day-clinic
- Outpatient
- Mixed setting
- Other (please specify): ______________

2. How many years of professional experience do you have in nutritional counselling in psychiatry?

- < 1 year
- 1–3 years
- 3–5 years
- 5–10 years
- 10+ years

3. Which qualification best applies to you?

- Dietitian (e.g., SVDE)
- Dietologist / dietetic assistant
- MSc in Nutritional Sciences
- Physician with a nutrition focus
- Other (please specify): ______________

Part 2 – Ratings

4. The following questions concern the qualities of a catering concept. How important to you are the following overarching goals of a catering concept in psychiatry?

(1 = not important … 5 = very important)

Item	1	2	3	4	5
Promotion of psychological stability					
Energy and nutrient adequacy					
Appetite appeal and eating enjoyment					
Metabolic health (e.g., reduction of metabolic side effects)					
Weight stabilisation / weight management					
Support of therapeutic interventions					
Sustainability / ecological aspects					
Cost-efficiency for the clinic					

5. Which specific needs of psychiatric patients should be particularly taken into account?

(1 = not important … 5 = very important)

Item	1	2	3	4	5
Loss of appetite / low eating motivation					

Hyperphagia / increased appetite					
Weight gain from psychotropic medication					
Metabolic changes from medication					
Eating disorders / restrictive eating					
Cognitive impairment					
Sensory sensitivity / overstimulation					
Cultural or religious requirements					
Other (please specify): ______________					

6. What role should the following dietary forms play in the new concept?

(1 = no role … 5 = important role)

Item	1	2	3	4	5
Mediterranean diet					
Wholefood diet (D-A-CH)					
Therapeutic diets (diabetes, lipid metabolism, etc.)					
Protein-rich diet					
Plant-based diet					
Low-carb / carbohydrate-reduced options					
Anti-inflammatory diet					
Individual special diets					

7. The following questions ask for your opinion on the design of the meals and the processes. Which characteristics of a meal are particularly important for psychiatric patients?

(1 = not important … 5 = very important)

Item	1	2	3	4	5
High satiety					
Clear structure / recognisable foods					
Short waiting times					
Simple choice options					
High taste palatability					
Visually appealing presentation					
Flexible portion sizes					
Beverage variety					
Low sugar and ultra-processing					

8. How important are the following organisational aspects?

(1 = not important … 5 = very important)

Item	1	2	3	4	5
Communication between nursing, kitchen, and dietetics					
Regular nutrition rounds					
Staff training in mealtime support					

Digital recording of food intake					
Option for individual nutrition plans					

9. Which barriers commonly exist in the current catering system?

(1 = does not apply at all … 5 = fully applies)

Item	1	2	3	4	5
Limited choice					
Insufficient quality / freshness					
Excessive energy density					
Insufficient personalisation					
Budget restrictions					
Difficult collaboration with the kitchen					
Other (please specify): ______________					

Part 3 – Open questions

10. In your view, which three aspects are most important for a new catering concept in psychiatry to be successful?

(Open free-text response.)

11. Which concepts or best practices from your experience should definitely be integrated?

(Open free-text response.)

12. In your view, are there risks that must be considered during the transition?

(Open free-text response.)

13. Do you have any further comments or recommendations?

(Open free-text response.)

Supplementary Checklist S1. Completed CHERRIES (Checklist for Reporting Results of Internet E-Surveys).

The completed checklist follows below.

Checklist for Reporting Results of Internet E-Surveys (CHERRIES)

Checklist Item	*Explanation*	**Authors' response**
Describe survey design	Describe target population, sample frame. Is the sample a convenience sample? (In "open" surveys this is most likely.)	Cross-sectional, anonymous, self-administered online expert survey of nutrition professionals working in psychiatric settings in the German-speaking D-A-CH region (Germany, Austria, and Switzerland). The sample was a non-probabilistic purposive/convenience sample. (Methods §2.1–2.2)
IRB approval	Mention whether the study has been approved by an IRB.	The study surveyed professionals and collected no patient or health-related personal data. Under the Swiss Federal Act on Research involving Human Beings (Human Research Act), such anonymous professional surveys fall outside the Act's scope and did not require approval by a cantonal ethics committee. The responsible cantonal ethics committee (Ethikkommission Nordwest- und Zentralschweiz, EKNZ) confirmed this in a formal clarification of responsibility (BASEC-ID Req-2026-00906, 7 July 2026). (Methods §2.7)
Informed consent	Describe the informed consent process. Where were the participants told the length of time of the survey, which data were stored and where and for how long, who the investigator was, and the purpose of the study?	Participation was voluntary and anonymous. On the introductory page, respondents were informed of the purpose and intended use of the survey and of the responsible institution (UPK Basel), and provided consent by proceeding with the survey. (Methods §2.2, §2.7)
Data protection	If any personal information was collected or stored, describe what mechanisms were used to protect unauthorized access.	The survey was anonymous and collected no identifying or health-related personal data. Responses were collected and stored on the Unipark platform (Tivian XI GmbH, Germany) and exported in de-identified form for analysis; as no personal data were captured, re-identification was not possible. (Methods §2.3, §2.7)
Development and testing	State how the survey was developed, including whether the usability and technical functionality of the electronic questionnaire had been tested before fielding the questionnaire.	The questionnaire was purpose-designed by the interdisciplinary project team (nutritional therapy, psychiatry, food services) on the basis of clinical experience and the literature; item wording and content were reviewed within the team for clarity and relevance. It was not formally validated. (Methods §2.3)
Open survey versus closed survey	An "open survey" is a survey open for each visitor of a site, while a closed survey is only open to a sample which the investigator knows (password-protected survey).	Open survey: distributed as an open link through professional networks; it was not password-protected or restricted to a closed, enumerated panel. (Methods §2.2)
Contact mode	Indicate whether or not the initial contact with the potential participants was made on the Internet. (Investigators may also send out questionnaires by mail and allow for Web-based data entry.)	Initial contact was made via the Internet: an invitation containing the survey link was disseminated through professional and clinical networks. (Methods §2.2)
Advertising the	How/where was the survey announced or	The survey was announced through professional and clinical networks—principally the ESSENzPSYCHE network for nutrition in psychiatry—(professional e-mail

survey	advertised? Some examples are offline media (newspapers), or online (mailing lists – If yes, which ones?) or banner ads (Where were these banner ads posted and what did they look like?). It is important to know the wording of the announcement as it will heavily influence who chooses to participate. Ideally the survey announcement should be published as an appendix.	contacts and distribution channels in dietetics and psychiatric nutritional care) across the German-speaking D-A-CH region. No public/offline media or banner advertisements were used. (Methods §2.2)
Web/E-mail	State the type of e-survey (eg, one posted on a Web site, or one sent out through e-mail). If it is an e-mail survey, were the responses entered manually into a database, or was there an automatic method for capturing responses?	Web-based survey hosted on the Unipark platform (Tivian XI GmbH, Germany); responses were captured automatically in the platform database. (Methods §2.3)
Context	Describe the Web site (for mailing list/newsgroup) in which the survey was posted. What is the Web site about, who is visiting it, what are visitors normally looking for? Discuss to what degree the content of the Web site could pre-select the sample or influence the results. For example, a survey about vaccination on a anti-immunization Web site will have different results from a Web survey conducted on a government Web site	The survey was not posted on a public website but distributed directly to nutrition professionals through professional networks, so website-based pre-selection does not apply. By design, the professional audience (dietitians, nutritional scientists, dietetic technicians, and physicians with a nutrition focus) constituted an expert sample. (Methods §2.2)
Mandatory/voluntary	Was it a mandatory survey to be filled in by every visitor who wanted to enter the Web site, or was it a voluntary survey?	Voluntary. (Methods §2.2)
Incentives	Were any incentives offered (eg, monetary, prizes, or non-monetary incentives such as an offer to provide the survey results)?	No incentives (monetary or non-monetary) were offered.
Time/Date	In what timeframe were the data collected?	16 February to 23 March 2026. (Methods §2.3)
Randomization of items or questionnaires	To prevent biases items can be randomized or alternated.	No. Items were presented in fixed thematic matrices; item order was not randomised or alternated. (Methods §2.3)
Adaptive questioning	Use adaptive questioning (certain items, or only conditionally displayed based on responses to other items) to reduce number and complexity of the questions.	No adaptive or conditional questioning was used; all respondents were presented with the same items. (Methods §2.3)

Number of Items	What was the number of questionnaire items per page? The number of items is an important factor for the completion rate.	44 rated items across six Likert matrices (overarching goals, 8; patient needs, 8; dietary forms, 8; meal characteristics, 9; organisational aspects, 5; barriers, 6), together with three background items and four open-ended questions. (Methods §2.3)
Number of screens (pages)	Over how many pages was the questionnaire distributed? The number of items is an important factor for the completion rate.	The questionnaire was organised into a background section, the six rating matrices, and an open-text section, presented over several screens. The exact number of screens was not recorded.
Completeness check	It is technically possible to do consistency or completeness checks before the questionnaire is submitted. Was this done, and if "yes", how (usually JAVAScript)? An alternative is to check for completeness after the questionnaire has been submitted (and highlight mandatory items). If this has been done, it should be reported. All items should provide a non-response option such as "not applicable" or "rather not say", and selection of one response option should be enforced.	No completeness check was enforced: response to individual items was not mandatory, so items could be left blank (item nonresponse was permitted). (Methods §2.3–2.4)
Review step	State whether respondents were able to review and change their answers (eg, through a Back button or a Review step which displays a summary of the responses and asks the respondents if they are correct).	Respondents could navigate between pages using the platform's standard controls. A dedicated review/summary step was not specifically implemented or recorded.
Unique site visitor	If you provide view rates or participation rates, you need to define how you determined a unique visitor. There are different techniques available, based on IP addresses or cookies or both.	Not determined. As an open survey distributed by link without site-visitor tracking, unique site visitors were not counted. (Methods §2.2)
View rate (Ratio of unique survey visitors/unique site visitors)	Requires counting unique visitors to the first page of the survey, divided by the number of unique site visitors (not page views!). It is not unusual to have view rates of less than 0.1 % if the survey is voluntary.	Could not be determined, because the number of unique site visitors (the denominator) was not available for this open, link-distributed survey. (Methods §2.2)
Participation rate (Ratio of unique visitors who agreed to participate/unique first survey page visitors)	Count the unique number of people who filled in the first survey page (or agreed to participate, for example by checking a checkbox), divided by visitors who visit the first page of the survey (or the informed consents page, if present). This can also be called "recruitment" rate.	Could not be meaningfully computed: the size of the invited population was unknown, as the survey was distributed openly through professional networks. (Methods §2.2)

Completion rate (Ratio of users who finished the survey/users who agreed to participate)	The number of people submitting the last questionnaire page, divided by the number of people who agreed to participate (or submitted the first survey page). This is only relevant if there is a separate "informed consent" page or if the survey goes over several pages. This is a measure for attrition. Note that "completion" can involve leaving questionnaire items blank. This is not a measure for how completely questionnaires were filled in. (If you need a measure for this, use the word "completeness rate".)	A precise completion rate could not be computed, as the number of people who opened the first page is not reliably known. Twenty-six responses were analysed; because items were optional, the valid n varied across items (24–26). (Methods §2.2, §2.4)
Cookies used	Indicate whether cookies were used to assign a unique user identifier to each client computer. If so, mention the page on which the cookie was set and read, and how long the cookie was valid. Were duplicate entries avoided by preventing users access to the survey twice; or were duplicate database entries having the same user ID eliminated before analysis? In the latter case, which entries were kept for analysis (eg, the first entry or the most recent)?	Cookies were not used to assign a unique user identifier or to prevent repeat participation.
IP check	Indicate whether the IP address of the client computer was used to identify potential duplicate entries from the same user. If so, mention the period of time for which no two entries from the same IP address were allowed (eg, 24 hours). Were duplicate entries avoided by preventing users with the same IP address access to the survey twice; or were duplicate database entries having the same IP address within a given period of time eliminated before analysis? If the latter, which entries were kept for analysis (eg, the first entry or the most recent)?	IP addresses were not used to identify or prevent duplicate entries.
Log file analysis	Indicate whether other techniques to analyze the log file for identification of multiple entries were used. If so, please describe.	No log-file analysis for the identification of multiple entries was performed.

Registration	In "closed" (non-open) surveys, users need to login first and it is easier to prevent duplicate entries from the same user. Describe how this was done. For example, was the survey never displayed a second time once the user had filled it in, or was the username stored together with the survey results and later eliminated? If the latter, which entries were kept for analysis (eg, the first entry or the most recent)?	Not applicable: this was an open survey with no login or registration.
Handling of incomplete questionnaires	Were only completed questionnaires analyzed? Were questionnaires which terminated early (where, for example, users did not go through all questionnaire pages) also analyzed?	Analyses were conducted item-wise on all available data; unanswered items were treated as missing and no imputation was performed, so the valid n varied across items (24–26). Partial responses were retained at the item level. A minor discrepancy between the analysed dataset (n = 26) and the field report (n = 27) is acknowledged. (Methods §2.4; Discussion §4.8)
Questionnaires submitted with an atypical timestamp	Some investigators may measure the time people needed to fill in a questionnaire and exclude questionnaires that were submitted too soon. Specify the timeframe that was used as a cut-off point, and describe how this point was determined.	No timestamp-based exclusion was applied; questionnaires were not excluded on the basis of completion time.
Statistical correction	Indicate whether any methods such as weighting of items or propensity scores have been used to adjust for the non-representative sample; if so, please describe the methods.	None. No weighting or propensity-score adjustment was applied; analyses were descriptive and exploratory, with no inferential testing. (Methods §2.5)

This checklist has been modified from Eysenbach G. Improving the quality of Web surveys: the Checklist for Reporting Results of Internet E-Surveys (CHERRIES). J Med Internet Res. 2004 Sep 29;6(3):e34 [erratum in J Med Internet Res. 2012; 14(1): e8.]. Article available at https://www.jmir.org/2004/3/e34/; erratum available https://www.jmir.org/2012/1/e8/. Copyright ©Gunther Eysenbach. Originally published in the Journal of Medical Internet Research, 29.9.2004 and 04.01.2012 [19].

This is an open-access article distributed under the terms of the Creative Commons Attribution License (https://creativecommons.org/licenses/by/2.0/), which permits unrestricted use, distribution, and reproduction in any medium, provided the original work, first published in the Journal of Medical Internet Research, is properly cited.

Table S3. Complete qualitative coding matrix: every free-text response to the four open-ended prompts, with the codes and themes assigned to it.

ID	Qualification / experience	Prompt	Original response (verbatim German)	English translation	Codes assigned	Theme(s)
**R01**	Dietitian; 1–3 years	P1 Success factors	- Kommunikation aller involvierten Anspruchsgruppen - Transparenz - Klarheit	Communication among all stakeholders involved; transparency; clarity.	Communication among all stakeholders; Transparency and clarity	T1 Interprofessional collaboration and leadership commitment
**R01**	Dietitian; 1–3 years	P2 Best practices	Genuss wird über alle Sinne angesprochen. Die Präsentation der Speise beginnt mit dem Menüplan, geht weiter auf dem Teller und über das Gericht entschieden wird spätestens nach der Nahrungsaufnahme. Essen/Trinken verbindet und berührt - Berührung braucht jeder Mensch und hilft zum Heilen.	Enjoyment is addressed through all the senses. The presentation of the dish begins with the menu plan, continues on the plate, and the dish is judged at the latest after it has been eaten. Eating and drinking connect and touch people — everyone needs to be touched, and it helps healing.	Enjoyment through all senses; Presentation from menu to plate; Eating connects and heals	T7 Eating enjoyment, palatability and dignity
**R01**	Dietitian; 1–3 years	P3 Perceived risks	Welche Grenzen (Budget, Aufwand etc.) werden aus der Leitung fix festgelegt? Wer sitzt schon alles zusammen in einem Boot?	Which limits (budget, workload, etc.) are fixed by management? Who is already sitting together in one boat?	Budget and workload limits set by management; Who is on board	T2 Economic, staffing and operational constraints; T1 Interprofessional collaboration and leadership commitment
**R02**	Dietitian; >10 years	P1 Success factors	Ausreichendes, vielseitiges à la Carte oder Einzelkomponentenangebot, wo alle Altersgruppen von 14 - 95 jährig berücksichtigt sind. Tägliche Mahlzeitenbestellung beim Patienten wie im Spital, dass die Pat. nach ihren Wünschen gefragt werden.	A sufficient, varied à la carte or single-component offering that caters for all age groups from 14 to 95. Daily meal ordering with the patient as in a general hospital, so that patients are asked about their wishes.	À la carte / single-component offering; Caters for all age groups; Patients asked daily about their wishes	T3 Choice, flexibility and individualisation of meals; T6 Patient and peer involvement
**R02**	Dietitian; >10 years	P3 Perceived risks	Pflege und Küche müssen mitmachen und sich für ihre Gäste / Klienten / Patienten interessieren	Nursing and the kitchen have to join in and take an interest in their guests / clients / patients.	Nursing and kitchen must join in	T1 Interprofessional collaboration and leadership commitment
**R02**	Dietitian; >10 years	P4 Further comments	Digitale Erfassung, was die Patienten gegessen haben wäre eigentlich super. Aber so wie es bei uns in der Psychiatrie aktuell läuft ist dies unmöglich mit diesen vielen offenen Stationsküchen und Kühlschränken, wo sich die Patienten 24 Stunden selbständig verpflegen können.	Digital recording of what patients have eaten would actually be excellent. But the way things currently run in our psychiatric setting this is impossible, with all these open ward kitchens and refrigerators where patients can feed themselves around the clock.	Digital intake recording unworkable; Open ward kitchens, 24-h self-catering	T2 Economic, staffing and operational constraints
**R03**	Dietitian; 5–10 years	P1 Success factors	- Flexibel - angepasst an verschiedene Bedürfnisse z.b. für ältere Mensche und Junge, zur Gewichtskontrolle oder fürs zunehmen - Ausgewogenheit -	Flexible; adapted to different needs, e.g. for older and younger people, for weight control or for gaining weight; balance.	Flexibility; Adapted to differing needs; Balanced provision	T3 Choice, flexibility and individualisation of meals
**R03**	Dietitian; 5–10 years	P2 Best practices	- ausreichend Proteine (für guete Sättigung) inkl. Wahlmöglichkeiten - angelehnt an die mediterrane Ernährung, angepasst ans schweizer Angebot	Enough protein (for good satiety), including choice options; based on Mediterranean eating, adapted to what is available in Switzerland.	Adequate protein for satiety; Mediterranean pattern adapted locally; Choice options	T5 Mediterranean, plant-forward provision with adequate protein; T3 Choice, flexibility and individualisation of meals
**R03**	Dietitian; 5–10	P3 Perceived	- gute Kommunikation mit Begründung (war bei uns wichtig, sonst gibts Gegenwind) - gut erkennbar,	Good communication, with reasons given (that mattered with us, otherwise you get pushback); clearly	Communication with reasons given;	T1 Interprofessional collaboration and

ID	Qualification / experience	Prompt	Original response (verbatim German)	English translation	Codes assigned	Theme(s)
	years	risks	welche Kostform für was geeignet ist.	recognisable which dietary form is suitable for what.	Pushback if imposed; Dietary forms clearly signposted	leadership commitment; T4 Staff training and nutrition literacy
**R04**	Dietitian; >10 years	P1 Success factors	Die interdisziplinäre Zusammenarbeit, intuitive Wahlmöglichkeiten, Gute Sättigung der Mahlzeiten (Ausgewogenheit)	Interdisciplinary collaboration; intuitive choice options; good satiety of the meals (balance).	Interdisciplinary collaboration; Intuitive choice options; Satiety and balance	T1 Interprofessional collaboration and leadership commitment; T3 Choice, flexibility and individualisation of meals
**R04**	Dietitian; >10 years	P2 Best practices	Mediterrane Kost, Vegetarische Kost ohne untrahochverarbeitete Proteinbeilagen, Vollkost mit fleischlosen Tagen zwingend Ernährungsberatung bei gewünschter veganer Kost (Schulung der Ausgewogenheit) und beim Wunsch zum weglassen von mehr als 5 Lebensmitteln	Mediterranean fare; vegetarian fare without ultra-processed protein sides; full diet with meat-free days. Dietetic counselling mandatory where a vegan diet is requested (training in balance) and where more than five foods are to be omitted.	Mediterranean fare; Vegetarian without ultra-processed protein sides; Meat-free days; Mandatory dietetic counselling when food groups omitted	T5 Mediterranean, plant-forward provision with adequate protein; T8 Risk of over-emphasising nutrition (pressure, disordered eating)
**R04**	Dietitian; >10 years	P3 Perceived risks	Obengenannte Wünsche zur Integration können mit wirtschaftlichen Herausforderungen kollidieren. Balance zwischen vielfältigen Kostformen und Personalmangel in der Verpflegung könnten ein Problem sein.	The wishes for integration mentioned above may collide with economic challenges. The balance between a variety of dietary forms and staff shortages in catering could be a problem.	Aspirations collide with economics; Variety vs. catering staff shortage	T2 Economic, staffing and operational constraints
**R05**	Dietitian; >10 years	P1 Success factors	Interdisziplinäre Zusammenarbeit, sowie Einbezug der Patientenrückmeldungen. Den Mut haben alte Gegebenheiten loszulassen und neue Erkenntisse /Systeme zu entwickeln. Gutes Schulungskonzept bei der Einführung.	Interdisciplinary collaboration, and incorporation of patient feedback. Having the courage to let go of old arrangements and to develop new insights and systems. A good training concept at introduction.	Interdisciplinary collaboration; Incorporating patient feedback; Courage to change; Training concept at rollout	T1 Interprofessional collaboration and leadership commitment; T6 Patient and peer involvement; T4 Staff training and nutrition literacy
**R05**	Dietitian; >10 years	P2 Best practices	Kostformenkatalog Ernährungspsychiatrie	A diet-forms catalogue; nutritional psychiatry.	Diet-forms catalogue; Nutritional psychiatry expertise	T4 Staff training and nutrition literacy
**R06**	Dietitian; 5–10 years	P1 Success factors	individuelle Auswahlmöglichkeit (würde auch individuelle Vorlieben und Bedürfnisse besser abdecken) möglichste hohe Qualität kleine feine Auswahl (evtl.. einzelne Komponenten wählbar)	Individual choice (this would also cover individual preferences and needs better); the highest possible quality; a small, refined selection (single components selectable if possible).	Individual choice; Covers individual preferences; Highest possible quality; Small refined selection	T3 Choice, flexibility and individualisation of meals; T7 Eating enjoyment, palatability and dignity
**R06**	Dietitian; 5–10 years	P2 Best practices	mehr Protein	More protein.	More protein	T5 Mediterranean, plant-forward provision with adequate protein
**R07**	Dietetic technician/assistant; >10 years	P1 Success factors	Bekannte Gerichte, guter Geschmack, Auswahlmöglichkeiten	Familiar dishes; good taste; choice options.	Familiar, recognisable dishes; Good taste; Choice options	T7 Eating enjoyment, palatability and dignity; T3 Choice,

ID	Qualification / experience	Prompt	Original response (verbatim German)	English translation	Codes assigned	Theme(s)
						flexibility and individualisation of meals
**R07**	Dietetic technician/assistant; >10 years	P2 Best practices	Buffet System, Aufklärung der Patienten	A buffet system; educating patients.	Buffet system; Patient education	T3 Choice, flexibility and individualisation of meals; T4 Staff training and nutrition literacy
**R07**	Dietetic technician/assistant; >10 years	P3 Perceived risks	Fehlende Kommunikation	A lack of communication.	Lack of communication	T1 Interprofessional collaboration and leadership commitment
**R08**	Dietitian; >10 years	P1 Success factors	Ausgewogenheit Möglichkeiten Mahlzeiten individuell anpassen zu können - durch Fachpersonal und bei Indikation Gute Kommunikation zwischen Küche und Ernährungsberatung	Balance; scope to adapt meals individually — by qualified staff and where indicated; good communication between the kitchen and dietetics.	Balance; Individual adaptation by qualified staff; Kitchen–dietetics communication	T3 Choice, flexibility and individualisation of meals; T1 Interprofessional collaboration and leadership commitment
**R08**	Dietitian; >10 years	P2 Best practices	Eine (begrenzte) Auswahlmöglichkeit für die Patienten, falls sie das Hauptmenu nicht mögen. Zuwahl von Zwischenmahlzeiten	A (limited) choice for patients if they do not like the main menu; the option to add snacks between meals.	Limited alternative to main menu; Optional between-meal snacks	T3 Choice, flexibility and individualisation of meals
**R08**	Dietitian; >10 years	P3 Perceived risks	Handling des Bestellsystems sollte einfach sein, da sonst die Fehlerquelle hoch. Falls bei dem System die Möglichkeit besteht Nährwertdaten der Mahlzeiten abzufragen, müsste gewährleistet sein, dass die Daten regelmässig kontrolliert und aktualisiert werden müssen.	Handling of the ordering system should be simple, otherwise the error rate is high. If the system allows nutrient data for meals to be retrieved, it would have to be guaranteed that the data are checked and updated regularly.	Ordering system must be simple; High error rate otherwise; Nutrient data must be maintained	T2 Economic, staffing and operational constraints
**R09**	Dietitian; 3–5 years	P1 Success factors	- Genügende finanzielle Resourecen - Interprofessionelle Zusammenarbeit -	Sufficient financial resources; interprofessional collaboration.	Sufficient financial resources; Interprofessional collaboration	T2 Economic, staffing and operational constraints; T1 Interprofessional collaboration and leadership commitment
**R10**	Other (nutritional science); 3–5 years	P1 Success factors	Befürwortung durch die Klinikleitung und Geschäftsführung Veränderungsbereitschaft der Küchenleitung und des Küchenpersonals Schulungen, Workshops o.ä. zur Veränderung der Küchenabläufe, Rezepte etc. ggf. durch externe Expert:innen	Endorsement by the clinical and commercial management; willingness to change on the part of the kitchen management and kitchen staff; training, workshops or similar to change kitchen processes, recipes etc., where appropriate by external experts.	Endorsement by clinical and commercial leadership; Kitchen willingness to change; Training and workshops, external experts	T1 Interprofessional collaboration and leadership commitment; T4 Staff training and nutrition literacy
**R11**	Dietitian; >10 years	P1 Success factors	- Integration von neuen ernährungswissenschaftlichen Erkenntnissen - Einfache Bedienbarkeit des Menüwahlsystems => Wer macht die Bestellung? und Umsetzung für die Küche - gute Auswahlmöglichkeiten für alle Mahlzeiten	Integration of new nutritional-science findings; simple operability of the menu-selection system — who places the order? and implementation for the kitchen; good choice for all meals.	Integrating new nutrition science; Usable ordering system; Implementable for	T4 Staff training and nutrition literacy; T2 Economic, staffing and operational constraints; T3

ID	Qualification / experience	Prompt	Original response (verbatim German)	English translation	Codes assigned	Theme(s)
					the kitchen; Good choice at all meals	Choice, flexibility and individualisation of meals
**R11**	Dietitian; >10 years	P2 Best practices	Mediterrane Kost	Mediterranean fare.	Mediterranean fare	T5 Mediterranean, plant-forward provision with adequate protein
**R11**	Dietitian; >10 years	P3 Perceived risks	Alle mit ins Boot nehmen, d.h. auch die Pflege sensiblisieren für die Wichtigkeit des Essens/-bestelung => allenfalls geschulte Hotellerie-Mitarbeiter:innen	Get everyone on board, i.e. sensitise nursing staff, too, to the importance of the meal and of meal ordering — possibly trained hospitality staff.	Get everyone on board; Sensitise nursing to the meal; Trained hospitality staff	T1 Interprofessional collaboration and leadership commitment; T4 Staff training and nutrition literacy
**R11**	Dietitian; >10 years	P4 Further comments	Ärzte und Pflege sensibilisieren für die Aspekte der unterstützenden Ernährung!	Sensitise physicians and nursing staff to the aspects of supportive nutrition!	Sensitise physicians and nursing	T4 Staff training and nutrition literacy
**R14**	Other (dietetic assistant, MSc); >10 years	P2 Best practices	Geduld, ankommen lassen, nicht überfordern, kurze und einfache Sätze auch für Muttersprachler, Speisekarten mit Foto, so wenig Text wie möglich und nötig, Schriftgröße mindestens 14, Zeilenabstand mindestens 1,5 Ernst nehmen, zuhören, alles beachten auch die Nebensätze	Patience; let things settle; do not overwhelm; short, simple sentences, including for native speakers; menus with photographs; as little text as possible and necessary; font size at least 14; line spacing at least 1.5. Take them seriously, listen, pay attention to everything — including the asides.	Do not overwhelm the patient; Photograph-based menus, minimal text, large type; Take patients seriously, listen	T7 Eating enjoyment, palatability and dignity; T6 Patient and peer involvement
**R14**	Other (dietetic assistant, MSc); >10 years	P3 Perceived risks	Personal könnte mit Unverständnis und Überforderung reagieren, denn das war doch immer so und richtig.	Staff might react with incomprehension and a sense of being overwhelmed, because this is how it has always been and it was right.	Staff incomprehension and resistance; Staff feel overwhelmed	T1 Interprofessional collaboration and leadership commitment; T2 Economic, staffing and operational constraints
**R14**	Other (dietetic assistant, MSc); >10 years	P4 Further comments	Ich würde trotz der gesunden Kost die gängigen Lieblingsgerichte aufnehmen und diese in angepasster Form zubereiten. Statt ganzer Pizza zum Abendessen gibt es ein mediterranes Teilstück, dazu Salat und Proteinbeilage. Pizza ist also nicht mehr die komplette Mahlzeit sondern ein ergänzender Bestandteil. Solche Beispiele können auch zu Hause in der Familie oder mit Freunden umgesetzt werden. Lerneffekte bei Mahlzeiten gehen durch den Magen und sollten zur Theorie passen.	Despite the healthy fare I would include the popular favourite dishes and prepare them in adapted form. Instead of a whole pizza for supper there is a Mediterranean slice, with salad and a protein side. Pizza is thus no longer the complete meal but a complementary component. Such examples can also be put into practice at home with family or friends. Learning at mealtimes goes through the stomach and should match the theory.	Keep favourite dishes in adapted form; Mediterranean portion with salad and protein; Learning through the meal itself	T5 Mediterranean, plant-forward provision with adequate protein; T7 Eating enjoyment, palatability and dignity
**R15**	Physician, nutrition focus; >10 years	P1 Success factors	Vermittlung der Effektivität von Ernährung als Therapie Vermittlung als Achtsamkeit insgesamt Multidisziplinäre Zusammenarbeit und Verständnis für das Thema	Conveying the effectiveness of nutrition as therapy; conveying it as mindfulness overall; multidisciplinary collaboration and understanding of the subject.	Conveying nutrition as therapy; Multidisciplinary collaboration and understanding	T4 Staff training and nutrition literacy; T1 Interprofessional collaboration and leadership commitment
**R15**	Physician, nutrition focus; >10 years	P2 Best practices	Ernährungsverhalten in Kombi mit individuellen psychologischen Aspekten Leistbarkeit Erreichbarkeit und Lagerungsmöglichkeiten	Eating behaviour combined with individual psychological aspects; affordability; accessibility and storage options.	Eating behaviour with individual psychology; Affordability; Accessibility and	T3 Choice, flexibility and individualisation of meals; T2 Economic, staffing and operational

ID	Qualification / experience	Prompt	Original response (verbatim German)	English translation	Codes assigned	Theme(s)
					storage	constraints
**R15**	Physician, nutrition focus; >10 years	P3 Perceived risks	Fixierung auf Ernährung als einzige Lösung - Standards für alle Fördert ev essstörungen	Fixation on nutrition as the only solution — standards for everyone. May promote eating disorders.	Nutrition as the only solution; Uniform standards for everyone; May promote eating disorders	T8 Risk of over-emphasising nutrition (pressure, disordered eating)
**R16**	Other; <1 year	P1 Success factors	Wissen um die hohe Bedeutung, die schmackhaftes, ausreichendes, ansehnlich angerichtetes Essen für das seelische Wohlbefinden hat Förderung der Genussfähigkeit KEINE zusätzlichen Verbote und Restriktionen, die für die Patienten weiteren Stress verursachen	Awareness of the great importance that tasty, sufficient, attractively presented food has for mental wellbeing; fostering the capacity for enjoyment; NO additional prohibitions and restrictions that cause patients further stress.	Tasty, sufficient, attractive food for mental wellbeing; Fostering capacity for enjoyment; No additional prohibitions or restrictions	T7 Eating enjoyment, palatability and dignity; T8 Risk of over-emphasising nutrition (pressure, disordered eating)
**R16**	Other; <1 year	P3 Perceived risks	Gesteigerter Druck auf Patienten Überbetonung der Bedeutung von Ernährungsdetails schafft eher Unsicherheit Freiräume ohne Überwachung und die Möglichkeit zum Genuss (inkl. Süßigkeiten) müssen erhalten bleiben	Increased pressure on patients. Over-emphasising the importance of dietary details tends to create insecurity. Unmonitored freedom and the possibility of enjoyment (including sweets) must be preserved.	Increased pressure on patients; Over-emphasis creates insecurity; Preserve unmonitored freedom and enjoyment	T8 Risk of over-emphasising nutrition (pressure, disordered eating); T7 Eating enjoyment, palatability and dignity
**R17**	MSc Nutritional Sciences; >10 years	P2 Best practices	Gesunde Ernährung mit Praxisschulungen, gute Kommunikation zwischen den Akteuren und hohe Ernährungskompetenz bzg. Psychischer Erkrankungen	Healthy nutrition with practical training, good communication between the actors involved, and high nutrition competence with respect to mental illness.	Practical training; Communication between actors; Nutrition competence in mental illness	T4 Staff training and nutrition literacy; T1 Interprofessional collaboration and leadership commitment
**R17**	MSc Nutritional Sciences; >10 years	P3 Perceived risks	Ernährung ist individuell und sehr komplex	Nutrition is individual and very complex.	Nutrition is individual and complex	T8 Risk of over-emphasising nutrition (pressure, disordered eating)
**R18**	MSc Nutritional Sciences; 1–3 years	P1 Success factors	Gute Kommunikation zu anderen Berufsgruppen Kostformkatalog zum Nachschlagen, um passende Kostform zu finden Umfangreiche Schulung zum Bestellsystem (z.B. für Pflege)	Good communication with other professional groups; a diet-forms catalogue to look things up in, so as to find the appropriate dietary form; thorough training on the ordering system (e.g. for nursing).	Communication with other professions; Diet-forms catalogue for reference; Thorough training on ordering system	T1 Interprofessional collaboration and leadership commitment; T4 Staff training and nutrition literacy
**R18**	MSc Nutritional Sciences; 1–3 years	P2 Best practices	Mehrere Kostformen für jeweilige Stoffwechsellage z. B. Ballaststoffreiche Ernährung	Several dietary forms for the respective metabolic situation, e.g. a high-fibre diet.	Dietary forms matched to metabolic status; High-fibre diet	T3 Choice, flexibility and individualisation of meals; T5 Mediterranean, plant-forward provision with adequate protein
**R18**	MSc Nutritional Sciences; 1–3 years	P3 Perceived risks	Unterversorgung vermeiden, viele ernährungsbedingte Erkrankungen im Blick behalten	Avoid undersupply; keep the many diet-related conditions in view.	Avoid undersupply; Keep diet-related conditions in view	T8 Risk of over-emphasising nutrition (pressure, disordered eating)

ID	Qualification / experience	Prompt	Original response (verbatim German)	English translation	Codes assigned	Theme(s)
**R19**	Other; >10 years	P1 Success factors	Tests bei Einweisung Intestinales Ökogramm Synlab Histamin, Tryptase, Allergien und Unverträglichkeiten Gentest, Personalisierte Ernährung, Nutrigenetik wenige gute Lebensmittel, einfache Kost Unverträglichkeiten wie Gluten, Lactose, Casain, Histamin... rausnehmen, dann gibt es keine Allergischen Reaktionen, keine Anaphylaxie mit Verwirrung oder Sepsis mehr in der Psychiatrie mit Haluzinationen durch Intoxikation von Lebensmitteln und Medikamenten	Tests on admission; intestinal ecogram (Synlab); histamine, tryptase, allergies and intolerances; genetic testing, personalised nutrition, nutrigenetics; few good foods, simple fare. Remove intolerances such as gluten, lactose, casein, histamine — then there are no more allergic reactions, no anaphylaxis with confusion or sepsis in psychiatry with hallucinations from intoxication by foods and medicines.	Universal testing on admission; Intestinal ecogram; Nutrigenetic testing; Food/medication 'intoxication' causing hallucinations, sepsis	Divergent case — coded but not incorporated into the thematic synthesis (Section 2.6)
**R19**	Other; >10 years	P2 Best practices	Nutrigenetic	Nutrigenetics.	Nutrigenetics	Divergent case — coded but not incorporated into the thematic synthesis (Section 2.6)
**R19**	Other; >10 years	P3 Perceived risks	Die Tests müssen bei Einweisung erfolgen, auf Grund falscher Ernährung kann es zum Herzstillstand kommen. Prof. Smollich - Studien, viele Patienten haben bereit Intoxikation durch Lebensmittel und Medikamenten und fehlende Tests, Sie laufen dann verwirrt herum, durch Gluten und Casain zum Beispiel...	The tests must be carried out on admission; incorrect nutrition can lead to cardiac arrest. Prof. Smollich — studies; many patients already have intoxication from foods and medicines and a lack of tests; they then walk around confused, from gluten and casein for example…	Testing on admission; Incorrect nutrition causing cardiac arrest; Food/medication 'intoxication'	Divergent case — coded but not incorporated into the thematic synthesis (Section 2.6)
**R19**	Other; >10 years	P4 Further comments	Test auf Autoimmunerkrankungen wie Hashimoto, Lupus, die meisten haben eine oder mehrere Autoimmunerkrankungen, die weder diagnostiziert noch behandelt werden. Bei vielen sieht man die deformierten Gelenke, denkt aber nicht, dass es lebensmittel- oder medikamenteninduziert ist. Oft werden die Allergien und Unverträglichkeiten sowie der Reizdarm, sowie die Autoimmunprozesse mit Schmerzen, Rheumaschübe u.a. vergessen. Die Patienten bilden sich die Erkrankung und Schmerzen nicht ein. Sie sind real. Leider fehlt die Expertise in neuro-immunologische Erkrankungen und Diagnostik. Umweltmediziner können hier sehr schnell und günstig helfen.	Testing for autoimmune diseases such as Hashimoto's, lupus; most have one or more autoimmune diseases that are neither diagnosed nor treated. In many one sees the deformed joints but does not think that it is food- or medication-induced. Allergies and intolerances, irritable bowel, and autoimmune processes with pain, rheumatic flares and so on are often forgotten. Patients are not imagining the illness and the pain. They are real. Unfortunately expertise in neuro-immunological disease and diagnostics is lacking. Environmental physicians can help here very quickly and cheaply.	Universal autoimmune testing; Food/medication-induced disease; Environmental medicine	Divergent case — coded but not incorporated into the thematic synthesis (Section 2.6)
**R20**	Other (BSc student, nutritional science); not reported	P1 Success factors	Betroffenensicht 1. Betroffene in das Versorgungskonzept mit einbeziehen (Möglichkeiten zur Mitgestaltung) 2. dieKliniken sollten die Möglichkeit haben, die Klinikverpflegung auch nach den Empfehlungen beispielsweise der DGE anzubieten. Die Diskrepanz zwischen dem wie man sich eigentlich ernähren sollte und dem was in vielen Kliniken budgetbedingt möglich ist unterstützt nicht gerade eine Ernährungsumstellung 3. um nachhaltig erfolgreich zu sein, sollte die Nachsorge nicht außer acht gelassen werden. Auch wenn eine Verbesserung während des Klinikaufenthaltes eintritt brauchen Betroffene anschließend noch weiterhin ihre Anlaufstellen. Wenn Betroffene nach ihrer Entlassung wieder in die selben Strukturen zurückkehren ohne	The perspective of those affected. (1) Involve those affected in the care concept (opportunities to help shape it). (2) Clinics should have the possibility of providing hospital catering in line with the recommendations of, for example, the DGE. The discrepancy between how one actually ought to eat and what is possible in many clinics for budgetary reasons hardly supports a change of diet. (3) To be successful in the long run, aftercare should not be neglected. Even if an improvement occurs during the hospital stay, those affected still need their points of contact afterwards. If they return after discharge to the same structures without these anchors, relapse will not be long in coming.	Involve those affected in the care concept; Budget prevents guideline-compliant catering; Aftercare and continuity	T6 Patient and peer involvement; T2 Economic, staffing and operational constraints

ID	Qualification / experience	Prompt	Original response (verbatim German)	English translation	Codes assigned	Theme(s)
			diese Anker, dann lässt ein Rückfall nicht lange auf sich warten			
**R20**	Other (BSc student, nutritional science); not reported	P2 Best practices	Patientenzentriert Interprofessionelle Zusammenarbeit Einbezug von Peerbegleitern und Angehörigen	Patient-centred; interprofessional collaboration; involvement of peer supporters and relatives.	Patient-centred; Interprofessional collaboration; Peer supporters and relatives	T6 Patient and peer involvement; T1 Interprofessional collaboration and leadership commitment
**R20**	Other (BSc student, nutritional science); not reported	P3 Perceived risks	Das größte Risiko ist, wenn eine der Professionen meint wichtiger zu sein als die andere.	The greatest risk is when one of the professions thinks it is more important than the other.	Professional hierarchy between disciplines	T1 Interprofessional collaboration and leadership commitment
**R20**	Other (BSc student, nutritional science); not reported	P4 Further comments	Danke für diese Studie!	Thank you for this study!	— (no substantive content)	No theme assigned (no substantive content)
**R21**	Other (dietetic management); >10 years	P1 Success factors	Verwaltung und Küche einbeziehen Parientenbefragung	Involve the administration and the kitchen; a patient survey.	Involve administration and kitchen; Patient survey	T1 Interprofessional collaboration and leadership commitment; T6 Patient and peer involvement
**R21**	Other (dietetic management); >10 years	P2 Best practices	Schulungen Workshops Selbstmanagement	Training; workshops; self-management.	Training and workshops; Self-management	T4 Staff training and nutrition literacy
**R21**	Other (dietetic management); >10 years	P3 Perceived risks	Zeitmanagement Kosten Personalmanagement	Time management; costs; staff management.	Time management; Costs; Staff management	T2 Economic, staffing and operational constraints
**R22**	Dietetic technician/assistant; >10 years	P1 Success factors	Alltagstauglich Pflanzlich basierend Auswahlmöglichkeiten	Suitable for everyday life; plant-based; choice options.	Suitable for everyday life; Plant-based; Choice options	T5 Mediterranean, plant-forward provision with adequate protein; T3 Choice, flexibility and individualisation of meals
**R22**	Dietetic technician/assistant; >10 years	P2 Best practices	Mediterrane Kost	Mediterranean fare.	Mediterranean fare	T5 Mediterranean, plant-forward provision with adequate protein
**R22**	Dietetic technician/assistant; >10 years	P3 Perceived risks	Eher nicht wir haben umgestellt, Patienten waren zufrieden. Schwierig ist es mit der kaufmännischen Klinikleitung und Vertretung	Not really — we made the change and patients were satisfied. What is difficult is the commercial clinic management and its representation.	Conversion went well, patients satisfied; Difficulty with commercial management	T1 Interprofessional collaboration and leadership commitment; T2 Economic, staffing and operational constraints
**R22**	Dietetic	P4 Further	Wir haben auf Station Ernährung DGE umgestellt	On the ward we converted to DGE-based nutrition.	Converted to	T5 Mediterranean,

ID	Qualification / experience	Prompt	Original response (verbatim German)	English translation	Codes assigned	Theme(s)
	technician/assistant; >10 years	comments	Vorteil Mehr regionale Produkte, pflanzliche Lebensmittel erhöht, Lebensmittel kosten gesenkt	Advantage: more regional products, more plant foods, food costs reduced.	guideline-based provision; More plant foods; Food costs reduced	plant-forward provision with adequate protein; T2 Economic, staffing and operational constraints
**R23**	Dietitian; 5–10 years	P1 Success factors	Auswahlmöglichkeiten für individuelle Bedürfnisse	Choice options for individual needs.	Choice for individual needs	T3 Choice, flexibility and individualisation of meals
**R23**	Dietitian; 5–10 years	P2 Best practices	entzündungsarme Ernährung, low carb Optionen	An anti-inflammatory diet; low-carbohydrate options.	Anti-inflammatory diet; Low-carbohydrate options	T5 Mediterranean, plant-forward provision with adequate protein
**R23**	Dietitian; 5–10 years	P3 Perceived risks	Gute Patienteninformation welche Kostform sinnvoll	Good patient information as to which dietary form makes sense.	Patient information on dietary forms	T6 Patient and peer involvement
**R25**	Physician, nutrition focus; >10 years	P1 Success factors	Mehr Wissen der Ärzte, Kooperation mit Küche, Verwaltung, kaufmännioscher Leitung: Klärung beispielsweise, dass hochwertige Ernährung sogar oft kostengünstiger: Linsengericht statt Hackfleisch /verschiertes laufende Unterstützung der Pflege bei Besprechungen mit den Patienten	More knowledge among physicians; cooperation with the kitchen, the administration and the commercial management: clarifying, for example, that high-quality nutrition is often even cheaper — a lentil dish instead of minced meat; ongoing support for nursing in discussions with patients.	More knowledge among physicians; Cooperation with kitchen, administration, commercial management; High quality can be cheaper; Ongoing support for nursing	T4 Staff training and nutrition literacy; T1 Interprofessional collaboration and leadership commitment; T2 Economic, staffing and operational constraints
**R25**	Physician, nutrition focus; >10 years	P2 Best practices	Basisschulung der Patienten durch entsprechnd geschulte Diätologen (die leider u.U. auch hochverarbeitete Wurstwaren oder Pressschinken für gesund halten!): Es besteht viel Unwissen, aber auch seitens des Krankenhauspersonals: daher Schulung vom Direktor bis zur Pfegehilfskraft. Entscheidend: nebst ein wenig fachlicher Information auch sinnvolle Beispiele für Einkauf und Speisenauswahl	Basic education of patients by appropriately trained dietitians (who unfortunately may themselves regard ultra-processed sausage or pressed ham as healthy!): there is a great deal of ignorance, including among hospital staff — hence training from the director down to the nursing assistant. Decisive: alongside a little technical information, also sensible examples for shopping and choosing dishes.	Basic patient education by trained dietitians; Ignorance among hospital staff; Training from director to nursing assistant; Practical shopping examples	T4 Staff training and nutrition literacy
**R25**	Physician, nutrition focus; >10 years	P3 Perceived risks	Personal einbeziehen, Widerstände hinterfragen, Sorgen oder Ängste ansprechen, um Widerstände zu reduzieren.	Involve staff, examine resistance, address worries or fears in order to reduce resistance.	Involve staff; Address resistance, worries and fears	T1 Interprofessional collaboration and leadership commitment
**R25**	Physician, nutrition focus; >10 years	P4 Further comments	Argumentieren Sie finanziell im Krankenhaus: qualitativ hochwertig kann auch sehr ökonomisch sein: bitte selbst Rechenbeispiele erstellen,	Argue financially in the hospital: high quality can also be very economical — please draw up your own worked examples.	Argue the business case; High quality can be economical	T2 Economic, staffing and operational constraints
**R26**	MSc Nutritional Sciences; 1–3 years	P1 Success factors	Verständnis und Anerkennung für die Bedeutung der Ernährung Monetäre Mittel Kommunikation	Understanding and recognition of the importance of nutrition; monetary resources; communication.	Recognition of the importance of nutrition; Monetary resources; Communication	T4 Staff training and nutrition literacy; T2 Economic, staffing and operational constraints; T1 Interprofessional collaboration and leadership commitment

ID	Qualification / experience	Prompt	Original response (verbatim German)	English translation	Codes assigned	Theme(s)
**R26**	MSc Nutritional Sciences; 1–3 years	P2 Best practices	Buffet, eigene Portionierung mit Anleitung Verteilung der Nährstoffe mittels Tellerprinzip	Buffet, self-portioning with guidance; distribution of nutrients using the plate model.	Buffet with guided self-portioning; Plate model for nutrient distribution	T3 Choice, flexibility and individualisation of meals; T5 Mediterranean, plant-forward provision with adequate protein
**R26**	MSc Nutritional Sciences; 1–3 years	P3 Perceived risks	Ausreichend Integration der Ernährungsdachkrfte im Prozess	Sufficient integration of the nutrition professionals in the process.	Integrate nutrition professionals in the process	T1 Interprofessional collaboration and leadership commitment
**R12**	MSc Nutritional Sciences; 1–3 years	—	No free-text response provided.	No free-text response provided.	—	—
**R13**	MSc Nutritional Sciences; 1–3 years	—	No free-text response provided.	No free-text response provided.	—	—
**R24**	Dietetic technician/assistant; 3–5 years	—	No free-text response provided.	No free-text response provided.	—	—

Prompts: P1 = the three most important success factors for the new meal concept; P2 = concepts or best practices that should be integrated; P3 = risks that must be considered during the change; P4 = further comments or recommendations. Responses are reproduced verbatim in the original German, including spelling and typing errors; the English translations were prepared for reporting. A response could be assigned to more than one theme, so the number of coded mentions (99) exceeds the number of responses (62 in the thematic synthesis). Theme prevalence in Figure 3 is the number of respondents contributing at least one coded extract to a theme. Free-text responses were provided by 23 of the 26 respondents; the responses of 22 entered the thematic synthesis, and those of R19 (shaded) were coded and reported as a divergent case in Section 3.9 without being incorporated into the synthesis. Themes: T1 = Interprofessional collaboration and leadership commitment; T2 = Economic, staffing and operational constraints; T3 = Choice, flexibility and individualisation of meals; T4 = Staff training and nutrition literacy; T5 = Mediterranean, plant-forward provision with adequate protein; T6 = Patient and peer involvement; T7 = Eating enjoyment, palatability and dignity; T8 = Risk of over-emphasising nutrition (pressure, disordered eating).

Supplementary Checklist S2. Completed COREQ (Consolidated Criteria for Reporting Qualitative Research) checklist for the qualitative component.

The 32-item COREQ checklist was developed for interview and focus-group studies. The qualitative component of the present study consists of written free-text answers to four open-ended questions embedded in an anonymous online survey; items that are specific to interviews and focus groups are therefore marked as not applicable, and the remaining items are answered for the free-text component. Section references are to the main text.

No. and item	Guide question / description	Authors' response
**Domain 1: Research team and reflexivity**		
**Personal characteristics**		
**1. Interviewer/facilitator**	Which author/s conducted the interview or focus group?	Not applicable. Data were collected by anonymous, self-administered online questionnaire; no interviews or focus groups were conducted.
**2. Credentials**	What were the researcher's credentials? E.g. PhD, MD.	No interviewer. Initial coding of the free-text responses was performed by T.L., a clinician-researcher at the study institution; codes, candidate themes and coded extracts were reviewed by the interdisciplinary co-author team (nutritional therapy, psychiatry and food services). Author credentials and roles are given in the affiliations and in the Author Contributions statement. (Methods §2.6)
**3. Occupation**	What was their occupation at the time of the study?	The analysis team comprised clinicians and nutrition professionals employed at the University Psychiatric Clinics Basel (UPK Basel) and the University of Basel, together with the hospital food-services management. (Methods §2.6; Reflexivity and positionality)
**4. Gender**	Was the researcher male or female?	Not reported.
**5. Experience and training**	What experience or training did the researcher have?	The project team combined clinical and research experience in psychiatry, nutritional therapy/dietetics and hospital food services. The coding followed the published six-phase reflexive thematic-analysis approach of Braun and Clarke. (Methods §2.6)
**Relationship with participants**		
**6. Relationship established**	Was a relationship established prior to study commencement?	No individual relationship was established. The survey link was distributed impersonally through professional and clinical networks, principally the ESSENzPSYCHE network for nutrition in psychiatry; participation was voluntary and anonymous. (Methods §2.2)
**7. Participant knowledge of the interviewer**	What did the participants know about the researcher? e.g. personal goals, reasons for doing the research	On the introductory page respondents were informed of the purpose and intended use of the survey and of the responsible institution (UPK Basel), namely the re-evaluation of its inpatient catering concept. No information about individual researchers was provided. (Methods §2.2, §2.7)
**8. Interviewer characteristics**	What characteristics were reported about the interviewer/facilitator? e.g. bias, assumptions, reasons and interests in the research topic	No interviewer. The analysts' insider position and prior commitments — including prior views about the value of a Mediterranean, plant-forward provision — are stated explicitly, together with the steps taken to mitigate confirmatory reading. (Methods §2.6, Reflexivity and positionality)
**Domain 2: Study design**		
**Theoretical framework**		
**9. Methodological orientation and theory**	What methodological orientation was stated to underpin the study? e.g. grounded theory, discourse analysis, ethnography, phenomenology, content analysis	Reflexive thematic analysis following the six-phase approach of Braun and Clarke, applied inductively and at the semantic level. The analysis is descriptive and codebook-like in orientation; no latent-level interpretation was attempted. (Methods §2.6)
**Participant selection**		
**10. Sampling**	How were participants selected? e.g. purposive, convenience, consecutive, snowball	Purposive and non-probabilistic (convenience) sampling of nutrition professionals with experience in, or professional relevance to, nutritional care in psychiatry, working predominantly in the German-speaking D-A-CH region. (Methods §2.2)
**11. Method of approach**	How were participants approached? e.g. face-to-face, telephone, mail, email	By an open invitation containing a link to the online questionnaire, disseminated through professional and clinical networks. (Methods §2.2)
**12. Sample size**	How many participants were in the study?	26 respondents provided complete questionnaires and constitute the analytic dataset. Free-text responses were provided by 23 respondents; the responses of 22 entered the thematic synthesis, and those of one further respondent were retained and reported as a divergent case. (Methods §2.2, §2.4; Results §3.9)
**13. Non-participation**	How many people refused to participate or dropped out? Reasons?	The platform recorded 71 accesses; 27 respondents answered the introductory background items and 26 submitted a completed questionnaire. All but one of the non-completers discontinued at or before the background section, so no demographic information is available for them; reasons for non-participation were

No. and item	Guide question / description	Authors' response
		not collected. (Methods §2.2)
**Setting**		
**14. Setting of data collection**	Where were the data collected? e.g. home, clinic, workplace	Online, at a time and place of the respondent's choosing, using the Unipark platform (Tivian XI GmbH, Cologne, Germany). (Methods §2.3)
**15. Presence of non-participants**	Was anyone else present besides the participants and researchers?	Not applicable to a self-administered online questionnaire; this could not be ascertained and was not controlled.
**16. Description of sample**	What are the important characteristics of the sample? e.g. demographic data, date	Work setting, years of experience in nutritional care in psychiatry and professional qualification are reported in Table 1 and Figure S6. The field period ran from 16 February to 23 March 2026. No further personal or demographic data were collected, consistent with the anonymous design. (Methods §2.3; Results §3.1)
**Data collection**		
**17. Interview guide**	Were questions, prompts, guides provided by the authors? Was it pilot tested?	Yes. The four open-ended prompts are reproduced in full, in the original German and in English translation, in Supplementary Questionnaire S1. The instrument was refined through iterative review within the interdisciplinary project team rather than by a separate external pilot. (Methods §2.3)
**18. Repeat interviews**	Were repeat interviews carried out? If yes, how many?	No. Each respondent completed the questionnaire once; the platform recorded no duplicate submissions. (Methods §2.4)
**19. Audio/visual recording**	Did the research use audio or visual recording to collect the data?	No. Responses were entered by respondents in writing and exported directly from the survey platform. (Methods §2.4)
**20. Field notes**	Were field notes made during and/or after the interview or focus group?	Not applicable; no interviews or focus groups were conducted and no field notes were made.
**21. Duration**	What was the duration of the interviews or focus group?	The time taken to complete the survey was not recorded in the platform configuration used. (Methods §2.3)
**22. Data saturation**	Was data saturation discussed?	Yes. Because the corpus was fixed and was coded in full, data saturation in the sense of iterative sampling does not apply; all responses were coded, and no themes beyond those present in the complete dataset could be generated. (Methods §2.6)
**23. Transcripts returned**	Were transcripts returned to participants for comment and/or correction?	No. The survey was anonymous, so respondents could not be recontacted. (Methods §2.2, §2.7)
**Domain 3: Analysis and findings**		
**Data analysis**		
**24. Number of data coders**	How many data coders coded the data?	Initial coding was performed by one analyst (T.L.) in the original German. The resulting codes, candidate themes and coded extracts were then reviewed by the interdisciplinary co-author team, and disagreements were resolved by discussion and by returning to the raw extracts. (Methods §2.6)
**25. Description of the coding tree**	Did authors provide a description of the coding tree?	Yes. The complete coding matrix — every free-text response with the codes and themes assigned to it — is provided as Table S3. Codes were kept close to respondents' own wording. (Methods §2.6; Table S3)
**26. Derivation of themes**	Were themes identified in advance or derived from the data?	Derived from the data. Coding was inductive and semantic; codes were generated from the responses rather than from an a priori framework. (Methods §2.6)
**27. Software**	What software, if applicable, was used to manage the data?	No dedicated qualitative-analysis software was used. Responses were exported from the Unipark platform and coded in a spreadsheet; the quantitative analyses and figures were produced in R version 4.3.3 with the ggplot2 package. (Methods §2.4, §2.5)
**28. Participant checking**	Did participants provide feedback on the findings?	No. Member checking was not possible because the survey was anonymous. (Methods §2.2)
**Reporting**		
**29. Quotations presented**	Were participant quotations presented to illustrate the themes/findings? Was each quotation identified? e.g. participant number	Yes. Illustrative extracts are quoted in Section 3.9 and in Table S2, each identified by respondent number (R01–R26) and, where informative, by qualification and years of experience. Extracts were translated from German for reporting; the original German wording is reproduced verbatim in Table S3. (Results §3.9; Tables S2, S3)
**30. Data and findings consistent**	Was there consistency between the data presented and the findings?	Yes. Every coded extract is traceable to its theme in Table S3, and theme prevalence in Figure 3 corresponds to the coding matrix. The thematic claims were additionally triangulated against the quantitative ratings, which were generated independently of the free-text analysis. (Methods §2.6; Results §3.9; Figure 3)
**31. Clarity of major themes**	Were major themes clearly presented in the findings?	Yes. The eight constructed themes are presented in Figure 3 and described in turn in Section 3.9, each with its

No. and item	Guide question / description	Authors' response
		respondent count and illustrative extracts. (Results §3.9)
**32. Clarity of minor themes**	Is there a description of diverse cases or discussion of minor themes?	Yes. Critical and divergent responses were deliberately retained and reported: the counterweight theme “Risk of over-emphasising nutrition” is described in full, and one respondent's cluster of non-evidence-based claims is reported separately as a divergent case and flagged as such in Table S3, without being incorporated into the thematic synthesis. (Methods §2.6; Results §3.9)

COREQ source: Tong, A.; Sainsbury, P.; Craig, J. Consolidated criteria for reporting qualitative research (COREQ): a 32-item checklist for interviews and focus groups. *Int. J. Qual. Health Care* 2007, 19, 349–357 [57].
